# SNP interactions of PGC with its neighbor lncRNAs enhance the susceptibility to gastric cancer/atrophic gastritis and influence the expression of involved molecules

**DOI:** 10.1002/cam4.1743

**Published:** 2018-08-28

**Authors:** Zhi Lv, Liping Sun, Qian Xu, Yuehua Gong, Jingjing Jing, Nannan Dong, Chengzhong Xing, Yuan Yuan

**Affiliations:** ^1^ Tumor Etiology and Screening Department of Cancer Institute and General Surgery China Medical University First Hospital Shenyang China; ^2^ The Key Laboratory of Cancer Etiology and Prevention Liaoning Provincial Education Department China Medical University Shenyang China

**Keywords:** atrophic gastritis, gastric cancer, LncRNA, PGC, polymorphism, susceptibility

## Abstract

Multidimensional interactions of multiple factors are more important in promoting cancer initiation. Gene‐gene interactions between protein‐coding genes have been paid great attention, while rare studies refer to the interactions between encoding and noncoding genes. Our research group previously found encoding gene PGC polymorphisms could affect the susceptibility to atrophic gastritis (AG) and gastric cancer (GC). Interestingly, several SNPs in long noncoding RNA (lncRNA) genes, just adjacent to PGC, were found to be associated with AG risk and GC prognosis afterward. This study aims to explore the SNP interactions between PGC and its neighbor lncRNAs on the risk of AG and GC. Genotyping for seven PGC SNPs and seven lncRNA SNPs was conducted using Sequenom MassARRAY platform in a total of 2228 northern Chinese subjects, including 536 GC cases, 810 AG cases, and 882 controls. We found 15 pairwise PGC‐lncRNAs SNPs had interactions: Five pairs were associated with AG risk, and ten pairs were associated with GC risk. Moreover, two GC‐related interactions PGC rs6939861 with lnc‐C6orf‐132‐1 rs7749023 and rs7747696 survived the Bonferroni correction (*P*_correction_ = 0.049 and 0.007, respectively). Several combinations showed obvious epistasis and cumulative effects on disease risk. Some three‐way interactions of SNPs with smoking and drinking could also be observed. Besides, a few interacting SNPs showed correlations with the expression levels of PGC protein and related lncRNAs in serum. Our study would provide research clues for further screening combination biomarkers uniting both protein‐coding and noncoding genes with the potential in prediction of the susceptibility to GC and its precursor.

## 1 INTRODUCTION

It has been extensively investigated that the most common form of genetic variation, single nucleotide polymorphisms (SNPs), can be potential biomarkers for risk prediction of cancer.^1^, ^2^, ^3^ However, the diagnostic efficacy for single SNP is limited, resulting from the multiple factors involved in carcinogenesis.^4^ A consensus has been reached that multidimensional interactions of various factors such as gene‐gene and gene‐environment are more important in promoting cancer initiation. Knowledge of gene‐gene and gene‐environment interactions could help to reveal substantial hidden heritability within the architecture of cancer susceptibility.^5^

In recent years, most investigations on gene‐gene interactions are mainly focused on encoding genes, while rare studies refer to the interactions between encoding and noncoding genes. As we know, among the sequences transcribed constantly in human genomes, only 1% are protein‐coding sequences and the vast majority is noncoding RNA (ncRNA).^6^ Currently, the number and types of known functional ncRNAs have increased considerably. A subset of both short‐ and long‐sized species are known to be involved in the regulation of target genes located at or near the same genomic locus. Their expression is often coordinated with that of nearby protein‐coding genes, and in many cases, related transcripts can influence each other at one step or another during their biogenesis.^7^ Therefore, exploration of interactions between encoding and their neighbor noncoding genes would be greatly beneficial for all round elucidation of gene impacts on physiological and disease states.

PGC protein, encoded by the pepsinogen C (PGC) gene, is a specific marker in the terminal differentiation of gastric mucosa, of which the aberrant expression occurs in many gastric diseases.^8^, ^9^, ^10^ Our research group previously found PGC polymorphisms could affect the susceptibility to atrophic gastritis (AG) and gastric cancer (GC).^11^, ^12^ Interestingly, several SNPs in long noncoding RNA (lncRNA) genes, just adjacent to PGC, were found to be associated with AG risk and GC prognosis afterward.^13^ However, it remains unclear whether PGC and its neighbor lncRNAs have SNP interactions with each other on the susceptibility to GC/AG.

In this study, we explored the SNP interactions between PGC and its neighbor lncRNAs on the risk of AG and GC, the modifying effects of environmental factors such as smoking, drinking, and *Helicobacter pylori* (*H. pylori*) infection, and the influence of SNP interactions on the expression of PGC protein and related lncRNAs. Our study aims to provide research clues for the identification of combination biomarkers uniting both protein‐coding and noncoding genes with the potential in risk prediction of GC and its precursor.

## 2 MATERIALS AND METHODS

### 2.1 Study subjects and epidemic‐clinical information collection

The study was approved by the Ethics Committee of China Medical University First Hospital. Written informed consent was obtained from all participants. A total of 2228 subjects were involved in our study, including 536 GC cases, 810 AG cases, and 882 controls. All enrolled individuals were recruited from the Zhuanghe Gastric Diseases Screening Program or hospitals in Zhuanghe and Shenyang of Liaoning Province, China between 2002 and 2013, which had been previously reported.^14^ The controls were frequency‐matched to the GC and AG cases, respectively, on the basis of gender and age (±5 years). That means an individual in the control group can be matched to an AG and a GC case simultaneously so long as it has the same sex both with them, and neither the age differences between the control and the AG nor the GC case are more than 5 years. Epidemiological data for each subject were obtained from face‐to‐face inquiry or the medical records of inpatients. After admission, gastroscopy examination was performed by experienced endoscopists. According to the updated Sydney system and the seventh edition of TNM staging,^15^, ^16^, ^17^ histopathological diagnoses were carried out independently by two gastrointestinal pathologists. Patients in the AG group were confirmed to have moderate to severe AG with or without intestinal metaplasia, and individuals confirmed to be with normal stomach or to have mild superficial gastritis were selected for the control group. Fasting venous blood samples (5 mL) were collected from each participant.

### 2.2 SNP selection

First, a two‐step approach was employed to select tagSNPs for PGC, which was described in our previous studies.^11^, ^12^ Then, we focused on the lncRNA genes nearby PGC using Ensembl genome browser,^13^ approximately encompassing 3 Mb of upstream and downstream flanking sequences of PGC. The selection of SNPs met the following criteria: (1) minor allele frequency (MAF) > 0.05 in CHB and JPT population, (2) pairwise linkage disequilibrium (*r* ^2^ < 0.8), and (3) according to Hardy‐Weinberg equilibrium (HWE, *P *>* *0.05). Consequently, seven PGC SNPs (rs6941539, rs9471643, rs6912200, rs6458238, rs3789210, rs4711690, and rs6939861) and seven lncRNA SNPs (rs7749023, rs7748341, rs7747696, rs72855279, and rs80112640 in lnc‐C6orf132‐1; rs1886753 in lnc‐LRFN2‐1; and rs61516247 in lnc‐LRFN2‐2) were selected as research targets.

### 2.3 Genotyping

Genomic DNA was extracted from each blood sample using phenol‐chloroform method. SNP genotyping was performed by Bio Miao Biological Technology (Beijing, China) applying Sequenom MassARRAY platform (Sequenom, San Diego, CA, USA). Additionally, we randomly selected 10% of the samples for repeated assays, and the results of all duplicated samples were 100% consistent.

### 2.4 Detection of *H*. *pylori*‐IgG titer, PGC protein, and lncRNAs in serum

The serum *H. pylori*‐IgG titer and PGC protein concentration were detected using an enzyme‐linked immunosorbent assay (ELISA kit, Biohit, Helsinki, Finland). Individuals with the titer > 34 IU were diagnosed as *H. pylori*‐positive. Total RNA was isolated from 400 μL of serum using a Blood Total RNA Isolation Kit (Bioteke, Beijing, China). Total RNA was converted into complementary DNA using a prime script RT master MIX (TaKaRa Biotech, Dalian, China). The lncRNA levels and an internal control gene, glyceraldehyde 3‐phosphate dehydrogenase (GAPDH), were examined using SYBR Premix Ex TaqII (TaKaRa Biotech, Dalian, China). Quantitative real‐time polymerase chain reaction (qRT‐PCR) was performed in an Eppendorf Mastercycler Gradient System (Eppendorf AG, Hamburg, Germany) according to the manufacturer's protocol. The sequences of primers used in qRT‐PCR were presented in Table S1. All the primers were synthesized by The Beijing Genomics Institute (Beijing, China). Melting curve analysis was performed to exclude the presence of nonspecific products and primer dimers. No template controls were included in each experiment. The relative quantification of lncRNA levels was calculated using the 2^−ΔCt^ method.

### 2.5 Statistical analysis

The differences in epidemiological characteristics between case and control groups were evaluated using the chi‐squared test. The multinomial logistic regression was applied to estimate the risk of gastric diseases by calculating odds ratios (ORs) with 95% confidence intervals (CIs). The log‐likelihood ratio test was employed to assess the interactions among PGC SNPs, lncRNA SNPs, and environmental factors by comparing the model that only contained the main effects of each factor with the full model that also contained interaction items. The ORs with 95% CIs were adjusted by gender, age, and *H. pylori* infection status unless *H. pylori* was regarded as an interaction item. The Cochran‐Armitage test for linear trend was used to judge the dosage effect on diseases risk with an increasing number of interacting factors. The difference in PGC protein and lncRNA levels in serum between two groups was compared using the Student's *t* test. The statistical analyses mentioned above were conducted using SPSS 22.0 software (SPSS, Chicago, IL, USA). All the tests were two‐sided, and *P* < 0.05 was considered to be statistically significant. The Bonferroni correction was used to adjust *P* values for multiple tests as needed. Additionally, the dominant, recessive, and overdominant models were defined as heterozygote+homozygote variant vs. homozygote wild, homozygote variant vs heterozygote+homozygote wild, and heterozygote vs. homozygote wild+homozygote variant, respectively.^18^

## 3 RESULTS

### 3.1 Baseline characteristics of the subjects

The study subjects consisted of 810 AG cases matched with 880 controls and 536 GC cases matched with 748 controls. No significant difference was found in the distribution of gender and age between the two pairwise groups of cases and controls (Table S2).

### 3.2 Association of single SNPs with AG and GC risk

A total of 14 SNPs were involved in the study, of which the relationship with the susceptibility to GC/AG had been previously investigated by our research group. Among the seven PGC polymorphisms, rs6458328 GA+AA genotype, rs3789210 CG+GG genotype, and rs4711690 CG+GG genotype were associated with a decreased AG risk (*P* = 0.015, OR = 0.73; *P* = 0.048, OR = 0.78; *P* = 0.008, OR = 0.78, respectively). The GC genotype of rs9471643 could reduce GC risk when compared with CC+GG genotype (*P* = 0.025, OR = 0.79). Furthermore, rs6939861 GA+AA genotype was linked to an increased risk of both AG and GC (AG: *P* = 0.014, OR = 1.30; GC: *P* = 0.015, OR = 1.32). However, no association with any disease risk was found in rs6941539 and rs6912200.^11^, ^12^ As for the seven lncRNA polymorphisms, rs61516247 was suggested to be associated with AG risk in overall population; while none was observed to have relationship with GC risk.^13^

### 3.3 Interactions between PGC and lncRNA SNPs on AG and GC risk

The SNP interactions of PGC with its neighbor lncRNAs were analyzed at first. Based on our previously published data, best genetic models with significant ORs in the main effect analysis were selected for each SNP. The results suggested five pairwise PGC‐lncRNA SNPs had negative interaction effects on AG risk, including rs9471643‐rs7749023 (*P*_interaction_ = 0.015, interaction index = 0.59), rs9471643‐rs7747696 (*P*_interaction_ = 0.018, interaction index = 0.60), rs6912200‐rs7749023 (*P*_interaction_ = 0.017, interaction index = 0.56), rs6912200‐rs7747696 (*P*_interaction_ = 0.031, interaction index = 0.60), and rs6912200‐rs1886753 (*P*_interaction_ = 0.023, interaction index = 0.54). For GC risk, ten pairwise PGC‐lncRNA SNPs showed interaction effects. Among them, eight combinations were positively interacted, which were rs6941539‐rs72855279 (*P*_interaction_ = 0.037, interaction index = 4.65), rs6941539‐rs80112640 (*P*_interaction_ = 0.038, interaction index = 4.60), rs6912200‐rs72855279 (*P*_interaction_ = 0.016, interaction index = 6.34), rs6912200‐rs80112640 (*P*_interaction_ = 0.017, interaction index = 6.27), rs6939861‐rs7749023 (*P*_interaction_ = 0.007, interaction index = 3.87), rs6939861‐rs7747696 (*P*_interaction_ = 0.001, interaction index = 4.88), rs6939861‐rs72855279 (*P*_interaction_ = 0.016, interaction index = 5.70), and rs6939861‐rs80112640 (*P*_interaction_ = 0.015, interaction index = 5.76), while the other two combinations, rs6941539‐rs7748341 (*P*_interaction_ = 0.007, interaction index = 0.48) and rs6939861‐rs61516247 (*P*_interaction_ = 0.041, interaction index = 0.40), were negatively interacted. Moreover, due to the large number of pairs tested, we used the Bonferroni correction to adjust *P* values for multiple comparison. And two GC‐related interactions rs6939861‐rs7749023 and rs6939861‐rs7747696 survived the correction (*P*_correction_ = 0.049 and 0.007, respectively), suggesting that they were strongly associated with GC risk (Table 1).

**Table 1:** The interaction effects between the SNPs in PGC and its neighbor lncRNAs on the risk of gastric diseases^a^

LncRNA SNP genotypes		PGC rs6941539		PGC rs9471643		PGC rs6912200		PGC rs6458238		PGC rs3789210		PGC rs4711690		PGC rs6939861	
		CC	CT+TT	GC	GG+CC	CC	CT+TT	GA+AA	GG	CG+GG	CC	CG+GG	CC	GG	GA+AA
AG vs. CON															
rs7749023															
AA	Case/control	330/403	131/118	159/217	304/304	101/131	362/393	64/97	401/426	109/141	357/384	178/232	287/292	176/234	274/265
	OR (95% CI)	1 (Ref)	1.35 (1.01‐1.80)	1 (Ref)	1.37 (1.06‐1.77)	1 (Ref)	1.20 (0.89‐1.62)	1 (Ref)	1.42 (1.01‐2.00)	1 (Ref)	1.20 (0.90‐1.60)	1 (Ref)	1.28 (1.00‐1.66)	1 (Ref)	1.39 (1.07‐1.80)
AC+CC	Case/control	221/228	115/120	135/124	203/224	99/85	239/261	46/55	294/294	65/72	275/277	145/162	195/186	135/143	193/189
	OR (95% CI)	1.17 (0.93‐1.48)	1.17 (0.88‐1.58)	1.48 (1.08‐2.04)	1.23 (0.93‐1.63)	1.51 (1.02‐2.23)	1.19 (0.87‐1.63)	1.21 (0.73‐2.01)	1.51 (1.06‐2.15)	1.15 (0.76‐1.75)	1.28 (0.94‐1.72)	1.16 (0.86‐1.56)	1.37 (1.03‐1.81)	1.25 (0.92‐1.70)	1.37 (1.03‐1.81)
		*P*_interaction_ = 0.367		***P***_**interaction**_ = **0.015** (**0.105** b **)**		***P***_**interaction**_ = **0.017** (**0.119** b **)**		*P*_interaction_ = 0.896		*P*_interaction_ = 0.642		*P*_interaction_ = 0.290		*P*_interaction_ = 0.181	
		Interaction index = 0.81		**Interaction index** = **0.59**		**Interaction index** = **0.56**		Interaction index = 0.96		Interaction index = 0.89		Interaction index = 0.80		Interaction index = 0.75	
rs7748341															
AA	Case/control	390/465	150/135	189/243	356/358	121/142	421/462	70/107	476/497	124/158	423/448	208/274	338/330	210/269	317/304
	OR (95% CI)	1 (Ref)	1.32 (1.01‐1.73)	1 (Ref)	1.28 (1.01‐1.63)	1 (Ref)	1.07 (0.81‐1.42)	1 (Ref)	1.46 (1.05‐2.02)	1 (Ref)	1.21 (0.92‐1.58)	1 (Ref)	1.36 (1.07‐1.72)	1 (Ref)	1.34 (1.06‐1.71)
AG+GG	Case/control	162/167	96/102	105/99	153/171	80/74	181/194	42/46	219/224	50/56	211/214	115/120	146/150	101/110	152/150
	OR (95% CI)	1.15 (0.89‐1.48)	1.13 (0.83‐1.54)	1.36 (0.97‐1.90)	1.15 (0.86‐1.54)	1.26 (0.85‐1.88)	1.10 (0.80‐1.51)	1.33 (0.79‐2.24)	1.49 (1.05‐2.13)	1.15 (0.73‐1.80)	1.26 (0.93‐1.70)	1.26 (0.92‐1.73)	1.29 (0.96‐1.72)	1.16 (0.84‐1.61)	1.31 (0.98‐1.75)
		*P*_interaction_ = 0.245		*P*_interaction_ = 0.067		*P*_interaction_ = 0.245		*P*_interaction_ = 0.544		*P*_interaction_ = 0.462		*P*_interaction_ = 0.127		*P*_interaction_ = 0.317	
		Interaction index = 0.76		Interaction index = 0.66		Interaction index = 0.75		Interaction index = 0.83		Interaction index = 0.82		Interaction index = 0.71		Interaction index = 0.79	
rs7747696															
AA	Case/control	303/382	119/113	145/206	281/290	95/127	329/372	59/91	368/408	106/132	322/368	165/225	262/275	156/221	256/254
	OR (95% CI)	1 (Ref)	1.32 (0.98‐1.78)	1 (Ref)	1.39 (1.06‐1.81)	1 (Ref)	1.20 (0.88‐1.62)	1 (Ref)	1.39 (0.97‐1.98)	1 (Ref)	1.09 (0.81‐1.46)	1 (Ref)	1.31 (1.01‐1.70)	1 (Ref)	1.44 (1.10‐1.88)
AG+GG	Case/control	249/250	127/128	148/136	229/243	105/92	274/285	52/62	328/317	68/84	312/296	159/173	221/205	155/158	214/204
	OR (95% CI)	1.24 (0.98‐1.56)	1.25 (0.94‐1.67)	1.55 (1.13‐2.12)	1.33 (1.01‐1.76)	1.53 (1.04‐2.25)	1.29 (0.94‐1.76)	1.24 (0.76‐2.04)	1.59 (1.10‐2.28)	0.99 (0.66‐1.50)	1.30 (0.96‐1.76)	1.25 (0.93‐1.67)	1.47 (1.11‐1.93)	1.37 (1.01‐1.86)	1.49 (1.12‐1.97)
		*P*_interaction_ = 0.482		***P***_**interaction**_ = **0.018(0.126** b **)**		***P***_**interaction**_ = **0.031** (**0.217** b **)**		*P*_interaction_ = 0.858		*P*_interaction_ = 0.538		*P*_interaction_ = 0.239		*P*_interaction_ = 0.121	
		Interaction index = 0.85		**Interaction index** = **0.60**		**Interaction index** = **0.60**		Interaction index = 1.05		Interaction index = 1.17		Interaction index = 0.78		Interaction index = 0.72	
rs72855279															
AA	Case/control	403/475	155/146	200/253	363/369	129/151	431/474	75/108	489/517	129/163	436/464	216/287	348/338	212/275	334/319
	OR (95% CI)	1 (Ref)	1.25 (0.96‐1.63)	1 (Ref)	1.24 (0.98‐1.57)	1 (Ref)	1.07 (0.81‐1.39)	1 (Ref)	1.37 (1.00‐1.89)	1 (Ref)	1.19 (0.91‐1.55)	1 (Ref)	1.37 (1.09‐1.73)	1 (Ref)	1.37 (1.08‐1.74)
AG+GG	Case/control	146/156	92/93	92/89	147/161	71/68	170/180	37/43	205/207	43/52	199/198	105/110	137/140	98/102	135/139
	OR (95% CI)	1.10 (0.85‐1.44)	1.18 (0.86‐1.61)	1.30 (0.92‐1.84)	1.16 (0.87‐1.55)	1.21 (0.81‐1.83)	1.11 (0.81‐1.53)	1.22 (0.72‐2.08)	1.45 (1.02‐2.06)	1.05 (0.66‐1.68)	1.27 (0.94‐1.73)	1.27 (0.92‐1.75)	1.31 (0.98‐1.76)	1.25 (0.90‐1.74)	1.28 (0.95‐1.72)
		*P*_interaction_ = 0.557		*P*_interaction_ = 0.167		*P*_interaction_ = 0.404		*P*_interaction_ = 0.954		*P*_interaction_ = 0.674		*P*_interaction_ = 0.105		*P*_interaction_ = 0.115	
		Interaction index = 0.87		Interaction index = 0.72		Interaction index = 0.81		Interaction index = 1.02		Interaction index = 0.89		Interaction index = 0.69		Interaction index = 0.69	
rs80112640															
AA	Case/control	403/474	155/146	201/253	360/369	129/151	431/474	73/108	489/516	129/163	434/363	216/287	346/338	214/275	330/318
	OR (95% CI)	1 (Ref)	1.25 (0.96‐1.63)	1 (Ref)	1.22 (0.97‐1.55)	1 (Ref)	1.07 (0.81‐1.39)	1 (Ref)	1.41 (1.02‐1.95)	1 (Ref)	1.19 (0.91‐1.55)	1 (Ref)	1.37 (1.08‐1.72)	1 (Ref)	1.35 (1.06‐1.71)
AG+GG	Case/control	149/157	92/95	93/89	148/164	72/68	172/183	38/45	206/208	44/53	200/200	108/111	136/142	97/104	139/139
	OR (95% CI)	1.12 (0.86‐1.45)	1.15 (0.84‐1.57)	1.31 (0.93‐1.85)	1.14 (0.86‐1.53)	1.23 (0.82‐1.85)	1.11 (0.81‐1.52)	1.23 (0.73‐2.09)	1.49 (1.04‐2.12)	1.06 (0.67‐1.68)	1.27 (0.94‐1.72)	1.29 (0.94‐1.78)	1.28 (0.96‐1.72)	1.20 (0.87‐1.67)	1.30 (0.97‐1.75)
		*P*_interaction_ = 0.514		*P*_interaction_ = 0.161		*P*_interaction_ = 0.346		*P*_interaction_ = 0.936		*P*_interaction_ = 0.686		*P*_interaction_ = 0.089		*P*_interaction_ = 0.221	
		Interaction index = 0.85		Interaction index = 0.72		Interaction index = 0.79		Interaction index = 0.98		Interaction index = 0.89		Interaction index = 0.68		Interaction index = 0.75	
rs1886753															
AG+GG	Case/control	386/470	177/177	213/255	353/391	134/170	433/476	78/122	491/526	112/157	458/493	236/290	334/358	221/283	332/340
	OR (95% CI)	1 (Ref)	1.22 (0.95‐1.56)	1 (Ref)	1.09 (0.86‐1.37)	1 (Ref)	1.17 (0.90‐1.52)	1 (Ref)	1.47 (1.08‐2.01)	1 (Ref)	1.31 (0.99‐1.72)	1 (Ref)	1.16 (0.92‐1.46)	1 (Ref)	1.26 (1.00‐1.59)
AA	Case/control	162/162	69/61	79/87	155/140	67/48	166/179	34/31	201/196	60/59	175/168	86/106	149/121	90/95	134/117
	OR (95% CI)	1.21 (0.94‐1.56)	1.38 (0.96‐2.00)	1.10 (0.77‐1.57)	1.32 (0.99‐1.77)	1.80 (1.16‐2.78)	1.18 (0.87‐1.61)	1.69 (0.96‐2.97)	1.62 (1.14‐2.29)	1.44 (0.93‐2.22)	1.46 (1.06‐2.01)	1.01 (0.72‐1.41)	1.51 (1.13‐2.03)	1.20 (0.85‐1.68)	1.48 (1.09‐2.01)
		*P*_interaction_ = 0.941		*P*_interaction_ = 0.448		***P***_**interaction**_ = **0.023** (**0.161** b **)**		*P*_interaction_ = 0.243		*P*_interaction_ = 0.525		*P*_interaction_ = 0.406		*P*_interaction_ = 0.886	
		Interaction index = 1.02		Interaction index = 0.83		**Interaction index** = **0.54**		Interaction index = 0.68		Interaction index = 0.84		Interaction index = 1.22		Interaction index = 1.04	
rs61516247															
GG	Case/control	258/325	110/111	140/181	234/255	88/102	286/335	50/77	327/363	73/106	304/334	154/197	223/243	148/196	212/220
	OR (95% CI)	1 (Ref)	1.25 (0.91‐1.70)	1 (Ref)	1.20 (0.90‐1.59)	1 (Ref)	1.01 (0.73‐1.40)	1 (Ref)	1.41 (0.95‐2.07)	1 (Ref)	1.33 (0.95‐1.87)	1 (Ref)	1.20 (0.91‐1.58)	1 (Ref)	1.29 (0.97‐1.71)
GA+AA	Case/control	294/307	137/130	154/161	277/278	113/117	317/322	62/76	370/362	101/110	332/330	170/201	262/237	163/183	258/238
	OR (95% CI)	1.21 (0.96‐1.52)	1.34 (1.00‐1.79)	1.26 (0.92‐1.72)	1.30 (0.98‐1.71)	1.15 (0.78‐1.69)	1.16 (0.84‐1.61)	1.26 (0.77‐2.06)	1.60 (1.09‐2.36)	1.35 (0.90‐2.02)	1.47 (1.05‐2.06)	1.10 (0.82‐1.48)	1.43 (1.09‐1.89)	1.18 (0.87‐1.60)	1.46 (1.10‐1.92)
		*P*_interaction_ = 0.591		*P*_interaction_ = 0.382		*P*_interaction_ = 0.839		*P*_interaction_ = 0.858		*P*_interaction_ = 0.846		*P*_interaction_ = 0.428		*P*_interaction_ = 0.927	
		Interaction index = 0.88		Interaction index = 0.83		Interaction index = 0.95		Interaction index = 0.95		Interaction index = 1.05		Interaction index = 1.18		Interaction index = 0.98	
GC vs. CON															
rs7749023															
CC	Case/control	25/27	9/19	9/12	25/34	12/13	22/33	13/11	21/35	5/9	29/37	13/16	21/30	19/17	14/28
	OR (95% CI)	1 (Ref)	0.51 (0.20‐1.34)	1 (Ref)	0.98 (0.36‐2.68)	1 (Ref)	0.72 (0.28‐1.87)	1 (Ref)	0.51 (0.19‐1.34)	1 (Ref)	1.41 (0.43‐4.67)	1 (Ref)	0.86 (0.34‐2.16)	1 (Ref)	0.45 (0.18‐1.12)
AC+AA	Case/control	360/512	137/181	173/272	326/422	128/166	367/528	79/122	421/575	128/169	372/529	201/313	299/384	181/303	299/363
	OR (95% CI)	0.76 (0.43‐1.33)	0.82 (0.45‐1.47)	0.85 (0.35‐2.06)	1.03 (0.43‐2.47)	0.84 (0.37‐1.89)	0.75 (0.34‐1.67)	0.55 (0.23‐1.28)	0.62 (0.28‐1.40)	1.36 (0.45‐4.17)	1.27 (0.42‐3.81)	0.79 (0.37‐1.68)	0.96 (0.45‐2.02)	0.53 (0.27‐1.06)	0.74 (0.38‐1.44)
		*P*_interaction_ = 0.086		*P*_interaction_ = 0.912		*P*_interaction_ = 0.733		*P*_interaction_ = 0.173		*P*_interaction_ = 0.565		*P*_interaction_ = 0.488		***P***_**interaction**_ = **0.007** (**0.049** b **)**	
		Interaction index = 2.47		Interaction index = 1.06		Interaction index = 1.20		Interaction index = 2.08		Interaction index = 0.69		Interaction index = 1.42		**Interaction index** = **3.87**	
rs7748341															
AA	Case/control	262/403	96/111	126/204	234/312	90/118	266/400	55/95	305/424	97/135	263/385	137/230	222/289	134/236	211/258
	OR (95% CI)	1 (Ref)	1.33 (0.97‐1.82)	1 (Ref)	1.21 (0.92‐1.61)	1 (Ref)	0.87 (0.64‐1.20)	1 (Ref)	1.24 (0.86‐1.79)	1 (Ref)	0.95 (0.70‐1.29)	1 (Ref)	1.29 (0.98‐1.70)	1 (Ref)	1.44 (1.09‐1.91)
AG+GG	Case/control	124/136	50/87	57/81	117/143	50/60	124/162	37/38	138/186	37/43	138/181	77/98	98/126	66/85	103/132
	OR (95% CI)	1.40 (1.05‐1.87)	0.88 (0.60‐1.29)	1.14 (0.76‐1.71)	1.33 (0.95‐1.84)	1.09 (0.69‐1.74)	1.00 (0.70‐1.44)	1.68 (0.96‐2.95)	1.28 (0.86‐1.91)	1.20 (0.72‐2.00)	1.06 (0.75‐1.49)	1.32 (0.92‐1.90)	1.31 (0.93‐1.83)	1.37 (0.93‐2.01)	1.37 (0.98‐1.92)
		***P***_**interaction**_ = **0.007 (0.049** b **)**		*P*_interaction_ = 0.881		*P*_interaction_ = 0.781		*P*_interaction_ = 0.118		*P*_interaction_ = 0.757		*P*_interaction_ = 0.155		*P*_interaction_ = 0.085	
		**Interaction index** = **0.48**		Interaction index = 0.96		Interaction index = 0.92		Interaction index = 0.60		Interaction index = 0.91		Interaction index = 0.69		Interaction index = 0.64	
rs7747696															
GG	Case/control	28/32	10/23	11/18	27/37	14/16	24/39	14/11	24/44	6/13	32/42	15/19	22/36	21/17	16/37
	OR (95% CI)	1 (Ref)	0.50 (0.20‐1.22)	1 (Ref)	1.19 (0.49‐2.94)	1 (Ref)	0.70 (0.29‐1.69)	1 (Ref)	0.43 (0.17‐1.09)	1 (Ref)	1.65 (0.57‐4.82)	1 (Ref)	0.77 (0.33‐1.83)	1 (Ref)	0.35 (0.15‐0.83)
AG+AA	Case/control	357/507	135/179	171/267	324/422	125/165	365/524	78/122	418/570	128/167	368/526	198/313	298/379	177/304	298/357
	OR (95% CI)	0.81 (0.48‐1.36)	0.86 (0.50‐1.50)	1.05 (0.48‐2.27)	1.26 (0.59‐2.70)	0.87 (0.41‐1.84)	0.80 (0.38‐1.65)	0.50 (0.22‐1.16)	0.58 (0.26‐1.28)	1.66 (0.61‐4.49)	1.52 (0.57‐4.03)	0.80 (0.40‐1.61)	1.00 (0.50‐1.99)	0.47 (0.24‐0.92)	0.68 (0.35‐1.30)
		*P*_interaction_ = 0.071		*P*_interaction_ = 0.845		*P*_interaction_ = 0.616		*P*_interaction_ = 0.079		*P*_interaction_ = 0.344		*P*_interaction_ = 0.294		***P***_**interaction**_ = **0.001** (**0.007** b **)**	
		Interaction index = 2.44		Interaction index = 0.91		Interaction index = 1.28		Interaction index = 2.49		Interaction index = 0.58		Interaction index = 1.64		**Interaction index** = **4.88**	
rs72855279															
GG	Case/control	10/9	5/13	2/6	13/16	9/5	6/17	6/4	9/18	3/5	12/17	6/9	9/13	9/8	6/14
	OR (95% CI)	1 (Ref)	0.35 (0.09‐1.36)	1 (Ref)	2.44 (0.42‐14.16)	1 (Ref)	0.20 (0.05‐0.82)	1 (Ref)	0.33 (0.08‐1.49)	1 (Ref)	1.18 (0.24‐5.89)	1 (Ref)	1.04 (0.27‐3.96)	1 (Ref)	0.38 (0.10‐1.47)
AG+AA	Case/control	376/529	141/187	181/279	339/440	131/176	384/543	87/127	434/595	131/174	390/549	208/322	312/400	191/311	308/380
	OR (95% CI)	0.64 (0.26‐1.59)	0.68 (0.27‐1.71)	1.95 (0.39‐9.75)	2.31 (0.46‐11.52)	0.41 (0.14‐1.26)	0.39 (0.13‐1.18)	0.46 (0.13‐1.67)	0.49 (0.14‐1.73)	1.26 (0.30‐5.35)	1.18 (0.28‐4.98)	0.97 (0.34‐2.76)	1.17 (0.41‐3.32)	0.55 (0.21‐1.44)	0.72 (0.28‐1.89)
		***P***_**interaction**_ = **0.037** (**0.259** b **)**		*P*_interaction_ = 0.446		***P***_**interaction**_ = **0.016** (**0.112** b **)**		*P*_interaction_ = 0.121		*P*_interaction_ = 0.861		*P*_interaction_ = 0.589		***P***_**interaction**_ = **0.016** (**0.112** b **)**	
		**Interaction index** = **4.65**		Interaction index = 0.49		**Interaction index** = **6.34**		Interaction index = 3.50		Interaction index = 0.86		Interaction index = 1.47		**Interaction index** = **5.70**	
rs80112640															
GG	Case/control	10/9	5/13	2/6	13/16	9/5	6/17	6/4	9/18	3/5	12/17	6/9	9/13	9/8	6/14
	OR (95% CI)	1 (Ref)	0.35 (0.09‐1.36)	1 (Ref)	2.44 (0.42‐14.16)	1 (Ref)	0.20 (0.05‐0.82)	1 (Ref)	0.33 (0.08‐1.49)	1 (Ref)	1.18 (0.24‐5.89)	1 (Ref)	1.04 (0.27‐3.96)	1 (Ref)	0.38 (0.10‐1.47)
AG+AA	Case/control	374/529	140/189	181/279	335/443	131/176	384/546	86/129	431/595	128/175	389/550	208/323	308/402	191/313	307/379
	OR (95% CI)	0.64 (0.26‐1.58)	0.67 (0.26‐1.68)	1.95 (0.39‐9.75)	2.27 (0.46‐11.31)	0.41 (0.14‐1.26)	0.39 (0.13‐1.18)	0.44 (0.12‐1.62)	0.48 (0.14‐1.72)	1.22 (0.29‐5.19)	1.18 (0.28‐4.96)	0.97 (0.34‐2.75)	1.15 (0.41‐3.26)	0.54 (0.21‐1.43)	0.72 (0.28‐1.89)
		***P***_**interaction**_ = **0.038** (**0.266** b **)**		*P*_interaction_ = 0.434		***P***_**interaction**_ = **0.017** (**0.119** b **)**		*P*_interaction_ = 0.116		*P*_interaction_ = 0.883		*P*_interaction_ = 0.604		***P***_**interaction**_ = **0.015** (**0.105** b **)**	
		**Interaction index** = **4.60**		Interaction index = 0.48		**Interaction index** = **6.27**		Interaction index = 3.56		Interaction index = 0.88		Interaction index = 1.45		**Interaction index** = **5.76**	
rs1886753															
GG	Case/control	77/119	34/51	48/69	65/101	23/45	87/125	17/34	96/137	25/42	88/129	52/69	61/102	42/74	64/91
	OR (95% CI)	1 (Ref)	1.03 (0.61‐1.73)	1 (Ref)	0.93 (0.57‐1.50)	1 (Ref)	1.36 (0.77‐2.41)	1 (Ref)	1.40 (0.74‐2.65)	1 (Ref)	1.15 (0.65‐2.02)	1 (Ref)	0.79 (0.49‐1.28)	1 (Ref)	1.24 (0.76‐2.03)
AG+AA	Case/control	308/420	112/149	135/216	286/356	117/135	302/437	76/99	346/475	109/138	313/437	162/262	259/312	157/246	250/302
	OR (95% CI)	1.13 (0.82‐1.56)	1.16 (0.80‐1.69)	0.90 (0.59‐1.38)	1.16 (0.77‐1.72)	1.70 (0.97‐2.97)	1.35 (0.80‐2.28)	1.54 (0.80‐2.95)	1.46 (0.80‐2.65)	1.33 (0.76‐2.31)	1.20 (0.72‐2.02)	0.82 (0.55‐1.24)	1.10 (0.74‐1.64)	1.12 (0.73‐1.73)	1.46 (0.96‐2.21)
		*P*_interaction_ = 0.703		*P*_interaction_ = 0.372		*P*_interaction_ = 0.112		*P*_interaction_ = 0.168		*P*_interaction_ = 0.499		*P*_interaction_ = 0.060		*P*_interaction_ = 0.986	
		Interaction index = 0.89		Interaction index = 1.30		Interaction index = 0.59		Interaction index = 0.59		Interaction index = 0.80		Interaction index = 1.72		Interaction index = 1.00	
rs61516247															
GA+GG	Case/control	353/499	138/187	169/266	325/423	128/163	361/526	86/125	409/567	121/164	374/529	199/310	295/382	179/301	295/361
	OR (95% CI)	1 (Ref)	1.04 (0.81‐1.35)	1 (Ref)	1.21 (0.95‐1.54)	1 (Ref)	0.87 (0.67‐1.14)	1 (Ref)	1.05 (0.78‐1.42)	1 (Ref)	0.96 (0.73‐1.26)	1 (Ref)	1.20 (0.95‐1.52)	1 (Ref)	1.37 (1.08‐1.75)
AA	Case/control	33/40	8/15	14/19	27/36	12/18	29/37	7/8	34/47	13/16	28/39	15/22	26/33	21/20	19/33
	OR (95% CI)	1.17 (0.72‐1.89)	0.75 (0.32‐1.80)	1.16 (0.57‐2.38)	1.18 (0.69‐2.02)	0.85 (0.40‐1.83)	1.00 (0.58‐1.71)	1.27 (0.45‐3.64)	1.05 (0.63‐1.77)	1.10 (0.51‐2.38)	0.97 (0.57‐1.67)	1.06 (0.54‐2.10)	1.23 (0.71‐2.11)	1.77 (0.93‐3.35)	0.97 (0.54‐1.75)
		*P*_interaction_ = 0.241		*P*_interaction_ = 0.666		*P*_interaction_ = 0.459		*P*_interaction_ = 0.828		*P*_interaction_ = 0.996		*P*_interaction_ = 0.874		***P***_**interaction**_ = **0.041** (**0.287** b **)**	
		Interaction index = 0.54		Interaction index = 0.82		Interaction index = 1.43		Interaction index = 0.88		Interaction index = 1.00		Interaction index = 0.93		**Interaction index** = **0.40**	

Notes

^a^ *P* for interaction was adjusted by gender, age, and *H. pylori* infection status; ^b^ *P* values after Bonferroni correction; AG, atrophic gastritis; GC, gastric cancer; CON, control; OR, odds ratio; CI, confidence interval. The results are in bold if *P* for interaction < 0.05.

### 3.4 Epistasis and cumulative effects of the interacting SNPs on AG and GC risk

We further examined the epistasis in the pairwise interacting SNPs. The results suggested when PGC rs6912200 CC genotype was present, lnc‐C6orf‐132‐1 rs7747696 AG+GG genotype conferred to a highest 1.79‐fold increased risk among AG‐related combinations (*P* = 0.006, OR = 1.79). Regarding the association with GC risk, when PGC rs6939861 GA+AA genotype was present, lnc‐C6orf‐132‐1 rs7747696 AG+AA genotype could elevate the risk most remarkably (*P* = 0.023, OR = 2.06, Table 2).

**Table 2:** The epistatic effects of the pairwise interacting SNPs on the risk of gastric diseases^a^

Pairwise interacting SNPs	Comparison	Subset	*P* (*P*_corr_)	OR (95% CI)
AG vs. CON				
PGC rs9471643 interacting with rs7749023	rs9471643 GG+CC vs. GC	rs7749023 AA	**0.004** (**0.020)**	**1.51** (**1.14**‐**1.99)**
		rs7749023 AC+CC	0.414	0.87 (0.63‐1.21)
	rs7749023 AC+CC vs. AA	rs9471643 GC	**0.014** (**0.070)**	**1.53** (**1.09**‐**2.13)**
		rs9471643 GG+CC	0.506	0.91 (0.70‐1.19)
PGC rs9471643 interacting with rs7747696	rs9471643 GG+CC vs. GC	rs7747696 AA	**0.003** (**0.015)**	**1.55** (**1.16**‐**2.07)**
		rs7747696 AG+GG	0.507	0.90 (0.66‐1.23)
	rs7747696 AG+GG vs. AA	rs9471643 GC	**0.006** (**0.030)**	**1.59** (**1.14**‐**2.22)**
		rs9471643 GG+CC	0.852	0.98 (0.75‐1.27)
PGC rs6912200 interacting with rs7749023	rs6912200 CT+TT vs. CC	rs7749023 AA	**0.025** (**0.125)**	**1.44** (**1.05**‐**1.99)**
		rs7749023 AC+CC	0.200	0.79 (0.56‐1.13)
	rs7749023 AC+CC vs. AA	rs6912200 CC	**0.008** (**0.040)**	**1.76** (**1.16**‐**2.68)**
		rs6912200 CT+TT	0.855	0.98 (0.77‐1.24)
PGC rs6912200 interacting with rs7747696	rs6912200 CT+TT vs. CC	rs7747696 AA	**0.026** (**0.130)**	**1.46** (**1.05**‐**2.03)**
		rs7747696 AG+GG	0.324	0.84 (0.60‐1.19)
	rs7747696 AG+GG vs. AA	rs6912200 CC	**0.006** (**0.030)**	**1.79** (**1.18**‐**2.71)**
		rs6912200 CT+TT	0.662	1.05 (0.83‐1.33)
PGC rs6912200 interacting with rs1886753	rs6912200 CT+TT vs. CC	rs1886753 AG+GG	0.083	1.28 (0.97‐1.68)
		rs1886753 AA	0.125	0.70 (0.44‐1.11)
	rs1886753 AA vs. AG+GG	rs6912200 CC	**0.015 (0.075)**	**1.78 (1.12**‐**2.82)**
		rs6912200 CT+TT	0.758	0.96 (0.74‐1.25)
GC vs. CON				
PGC rs6941539 interacting with rs7748341	rs6941539 CT+TT vs. CC	rs7748341 AA	0.091	1.32 (0.96‐1.83)
		rs7748341 AG+GG	**0.037** (**0.370)**	**0.62** (**0.40**‐**0.97)**
	rs7748341 AG+GG vs. AA	rs6941539 CC	**0.033** (**0.330)**	**1.39** (**1.03**‐**1.87)**
		rs6941539 CT+TT	0.064	0.65 (0.41‐1.03)
PGC rs6941539 interacting with rs72855279	rs6941539 CT+TT vs. CC	rs72855279 GG	**0.030** (**0.300)**	**0.11** (**0.02**‐**0.81)**
		rs72855279 AG+AA	0.608	1.07 (0.82‐1.40)
	rs72855279 AG+AA vs. GG	rs6941539 CC	0.171	0.52 (0.20‐1.33)
		rs6941539 CT+TT	0.136	2.28 (0.77‐6.74)
PGC rs6941539 interacting with rs80112640	rs6941539 CT+TT vs. CC	rs80112640 GG	**0.030** (**0.300)**	**0.11** (**0.02**‐**0.81)**
		rs80112640 AG+AA	0.668	1.06 (0.81‐1.38)
	rs80112640 AG+AA vs. GG	rs6941539 CC	0.166	0.52 (0.20‐1.32)
		rs6941539 CT+TT	0.144	2.24 (0.76‐6.62)
PGC rs6912200 interacting with rs72855279	rs6912200 CT+TT vs. CC	rs72855279 GG	**0.026** (**0.260)**	**0.16** (**0.03**‐**0.81)**
		rs72855279 AG+AA	0.859	0.98 (0.75‐1.28)
	rs72855279 AG+AA vs. GG	rs6912200 CC	0.095	0.38 (0.12‐1.18)
		rs6912200 CT+TT	0.094	2.31 (0.87‐6.12)
PGC rs6912200 interacting with rs80112640	rs6912200 CT+TT vs. CC	rs80112640 GG	**0.026** (**0.260)**	**0.16** (**0.03**‐**0.81)**
		rs80112640 AG+AA	0.820	0.97 (0.74‐1.27)
	rs80112640 AG+AA vs. GG	rs6912200 CC	0.095	0.38 (0.12‐1.18)
		rs6912200 CT+TT	0.097	2.29 (0.86‐6.07)
PGC rs6939861 interacting with rs7749023	rs6939861 GA+AA vs. GG	rs7749023 CC	**0.037** (**0.370)**	**0.35** (**0.13**‐**0.94)**
		rs7749023 AC+AA	**0.004** (**0.040)**	**1.44** (**1.12**‐**1.84)**
	rs7749023 AC+AA vs. CC	rs6939861 GG	**0.028** (**0.280)**	**0.45** (**0.22**‐**0.92)**
		rs6939861 GA+AA	0.098	1.78 (0.90‐3.50)
PGC rs6939861 interacting with rs7747696	rs6939861 GA+AA vs. GG	rs7747696 GG	**0.007** (**0.070)**	**0.27** (**0.11**‐**0.71)**
		rs7747696 AG+AA	**0.002** (**0.020)**	**1.48** (**1.16**‐**1.90)**
	rs7747696 AG+AA vs. GG	rs6939861 GG	**0.012** (**0.120)**	**0.42 (0.21**‐**0.83)**
		rs6939861 GA+AA	**0.023 (0.230)**	**2.06 (1.11**‐**3.85)**
PGC rs6939861 interacting with rs72855279	rs6939861 GA+AA vs. GG	rs72855279 GG	0.069	0.19 (0.03‐1.14)
		rs72855279 AG+AA	**0.011 (0.110)**	**1.37 (1.08**‐**1.74)**
	rs72855279 AG+AA vs. GG	rs6939861 GG	0.086	0.42 (0.15‐1.13)
		rs6939861 GA+AA	0.084	2.41 (0.89‐6.54)
PGC rs6939861 interacting with rs80112640	rs6939861 GA+AA vs. GG	rs80112640 GG	0.069	0.19 (0.03‐1.14)
		rs80112640 AG+AA	**0.009** (**0.090)**	**1.38** (**1.08**‐**1.76)**
	rs80112640 AG+AA vs. GG	rs6939861 GG	0.085	0.42 (0.15‐1.13)
		rs6939861 GA+AA	0.083	2.42 (0.89‐6.58)
PGC rs6939861 interacting with rs61516247	rs6939861 GA+AA vs. GG	rs61516247 GA+GG	**0.007** (**0.070)**	**1.41** (**1.10**‐**1.81)**
		rs61516247 AA	0.142	0.52 (0.22‐1.24)
	rs61516247 AA vs. GA+GG	rs6939861 GG	0.061	1.88 (0.97‐3.63)
		rs6939861 GA+AA	0.324	0.74 (0.41‐1.35)

Notes

^a^ *P* was adjusted by gender, age, and *H. pylori* infection status; *P*_corr_, *P* values after Bonferroni correction; AG, atrophic gastritis; GC, gastric cancer; CON, control; OR, odds ratio; CI, confidence interval. The results are in bold if *P* < 0.05.

To further evaluate the diagnostic efficacy of these two‐way combinations, we calculated the cumulative ORs for them. The samples were divided into three subgroups according to the number of interacting SNPs that individuals carried with. Four pairwise PGC‐lncRNA SNPs showed significant dosage effects. Three of them conferred to an elevated GC risk with the increasing number of risk genotypes, including rs6939861‐rs7747696 (*P*_trend_ = 0.043), rs6939861‐rs72855279 (*P*_trend_ = 0.048), and rs6939861‐rs80112640 (*P*_trend_ = 0.044); only rs6939861‐rs61516247 had a contrary effect on GC risk (*P*_trend_ = 0.049, Table 3).

**Table 3:** The cumulative effects of the pairwise interacting SNPs on the risk of gastric diseases^a^

Number of interacting SNP genotypes (PGC‐lncRNA)	Case	Control	*P*	OR (95% CI)
AG vs. CON				
rs9471643‐rs7749023				
0	159	217		1 (Ref)
1	439	428	0.002	1.52 (1.17‐1.97)
2	203	224	0.055	1.33 (0.99‐1.79)
			*P*_trend_ = 0.167	
rs9471643‐rs7747696				
0	145	206		1 (Ref)
1	429	426	0.001	1.56 (1.19‐2.04)
2	229	243	0.014	1.45 (1.08‐1.95)
			*P*_trend_ = 0.067	
** **rs6912200‐rs7749023				
0	101	131		1 (Ref)
1	461	478	0.011	1.50 (1.10‐2.05)
2	239	261	0.074	1.35 (0.97‐1.88)
			*P*_trend_ = 0.468	
rs6912200‐rs7747696				
0	95	127		1 (Ref)
1	434	464	0.010	1.52 (1.10‐2.10)
2	274	285	0.024	1.46 (1.05‐2.04)
			*P*_trend_ = 0.182	
rs6912200‐rs1886753				
0	134	170		1 (Ref)
1	500	524	0.044	1.32 (1.01‐1.74)
2	166	179	0.180	1.26 (0.90‐1.76)
			*P*_trend_ = 0.330	
GC vs. CON				
** **rs6941539‐rs7748341				
0	262	403		1 (Ref)
1	220	247	0.016	1.36 (1.06‐1.74)
2	50	87	0.451	0.86 (0.58‐1.27)
			*P*_trend_ = 0.433	
rs6941539‐rs72855279				
0	10	9		1 (Ref)
1	381	542	0.161	0.51 (0.20‐1.31)
2	141	187	0.310	0.61 (0.24‐1.58)
			*P*_trend_ = 0.848	
rs6941539‐rs80112640				
0	10	9		1 (Ref)
1	379	542	0.157	0.51 (0.20‐1.30)
2	140	189	0.293	0.60 (0.23‐1.55)
			*P*_trend_ = 0.924	
** **rs6912200‐rs72855279				
0	9	5		1 (Ref)
1	137	193	0.079	0.36 (0.12‐1.13)
2	384	543	0.062	0.34 (0.11‐1.05)
			*P*_trend_ = 0.493	
** **rs6912200‐rs80112640				
0	9	5		1 (Ref)
1	137	193	0.079	0.36 (0.12‐1.13)
2	384	546	0.061	0.34 (0.11‐1.05)
			*P*_trend_ = 0.467	
rs6939861‐rs7749023				
0	19	17		1 (Ref)
1	195	331	0.024	0.45 (0.22‐0.90)
2	299	363	0.245	0.66 (0.33‐1.32)
			*P*_trend_ = 0.066	
rs6939861‐rs7747696				
0	21	17		1 (Ref)
1	193	341	0.009	0.40 (0.20‐0.80)
2	298	357	0.180	0.63 (0.32‐1.24)
			***P***_**trend**_ = **0.043** (**0.430** b **)**	
rs6939861‐rs72855279				
0	9	8		1 (Ref)
1	197	325	0.078	0.41 (0.15‐1.11)
2	308	380	0.291	0.59 (0.22‐1.57)
			***P***_**trend**_ = **0.048** (**0.480** b **)**	
** **rs6939861‐rs80112640				
0	9	8		1 (Ref)
1	197	327	0.077	0.41 (0.15‐1.10)
2	307	379	0.287	0.59 (0.22‐1.57)
			***P***_**trend**_ = **0.044** (**0.440** b **)**	
** **rs6939861‐rs61516247				
0	179	301		1 (Ref)
1	316	381	0.004	1.43 (1.12‐1.83)
2	19	33	0.880	1.05 (0.57‐1.93)
			***P***_**trend**_ = **0.049** (**0.490** b **)**	

Notes

^a^ *P* was adjusted by gender, age, and *H. pylori* infection status; ^b^ *P* values after Bonferroni correction; AG, atrophic gastritis; GC, gastric cancer; CON, control; OR, odds ratio; CI, confidence interval. The results are in bold if *P* for trend < 0.05.

### 3.5 Interactions of three dimensions among the SNPs and environmental factors

We next investigated the interaction effects of three dimensions among the interacting PGC‐lncRNA SNPs and environmental factors, including *H. pylori* infection, smoking, and drinking. For AG risk, two combinations demonstrated positive interactions with smoking, which were rs9471643‐rs7749023 (*P*_interaction_ = 0.045, interaction index = 3.19) and rs9471643‐rs7747696 (*P*_interaction_ = 0.020, interaction index = 3.81, Table S3). Negative interaction was found between rs6941539‐rs7738341 and drinking on GC risk (*P*_interaction_ = 0.049, interaction index = 0.17, Table S4). However, no significance was observed in any three‐way combination of PGC SNP‐lncRNA SNP‐*H. pylori* on diseases risk (*P*_interaction_ > 0.05, Table S5).

The cumulative ORs of the three‐way interacting combinations for disease risk were also calculated. No significant dosage effect was indicated in them (Table S6).

### 3.6 Correlations of the interacting SNPs with PGC protein expression levels

To explore the possible mechanism in the SNP interactions of PGC with its neighbor lncRNAs, we analyzed the influence of the interacting SNPs on PGC protein expression in serum. Among AG‐related combinations, PGC rs6912200 CT+TT genotype showed significant lower PGII concentration than CC genotype in both total subjects and controls when lnc‐C6orf132‐1 rs7749023 and rs7747696 had AA genotype (rs6912200‐rs7749023 in total: *P* = 0.027; rs6912200‐rs7749023 in control: *P* = 0.013; rs6912200‐rs7747696 in total: *P* = 0.021; and rs6912200‐rs7747696 in control: *P* = 0.014, Table 4).

**Table 4:** The correlations between the pairwise interacting SNPs and PGC protein expression levels in serum

PGC‐lncRNA SNP genotypes		Total			Case			Control		
		N	Median (25%, 75%)	*P* (*P*_corr_)	N	Median (25%, 75%)	*P*	N	Median (25%, 75%)	*P* (*P*_corr_)
AG vs. CON										
rs9471643‐rs7749023										
GC	AA	375	8.8 (5.7, 15.5)		158	12.3 (6.6, 19.3)		217	8.2 (5.3, 12.2)	
GC	AC+CC	257	9.3 (6.0, 16.3)	0.504	133	10.5 (6.9, 18.4)	0.918	124	8.5 (5.4, 14.1)	0.758
GG+CC	AA	602	8.8 (6.1, 15.2)	0.806	302	11.8 (6.7, 19.2)	0.756	300	7.7 (5.6, 10.8)	0.129
GG+CC	AC+CC	426	9.2 (5.7, 17.5)	0.259	203	12.9 (7.3, 21.3)	0.084	223	7.3 (4.8, 11.7)	0.451
rs9471643‐rs7747696										
GC	AA	350	8.8 (5.7, 15.3)		144	12.2 (6.5, 19.4)		206	8.2 (5.5, 12.3)	
GC	AG+GG	283	9.3 (6.1, 16.3)	0.724	147	11.2 (6.9, 18.5)	0.904	136	8.2 (5.3, 12.9)	0.811
GG+CC	AA	566	8.7 (6.1, 14.8)	0.663	279	11.4 (6.6, 18.3)	0.814	287	7.7 (5.6, 10.8)	0.099
GG+CC	AG+GG	470	9.4 (5.7, 17.5)	0.326	229	13.1 (7.4, 21.2)	0.120	241	7.3 (4.9, 11.8)	0.352
rs6912200‐rs7749023										
CC	AA	231	9.5 (6.0, 15.8)		100	13.1 (7.2, 19.9)		131	8.6 (5.7, 13.3)	
CC	AC+CC	182	10.4 (6.2, 18.1)	0.952	97	11.8 (8.5, 21.3)	0.634	85	7.9 (5.2, 12.0)	0.239
CT+TT	AA	749	8.7 (6.1, 15.0)	**0.027** (**0.135)**	360	11.6 (6.6, 19.0)	0.267	389	7.7 (5.5, 10.6)	**0.013** (**0.065)**
CT+TT	AC+CC	499	9.1 (5.7, 16.5)	0.431	239	12.1 (6.7, 20.3)	0.941	260	7.5 (5.1, 12.4)	0.144
rs6912200‐rs7747696										
CC	AA	221	10.0 (6.0, 16.0)		94	13.1 (7.2, 19.8)		127	8.6 (5.7, 13.3)	
CC	AG+GG	196	10.4 (6.3, 18.1)	0.859	104	11.7 (8.2, 21.1)	0.773	92	8.0 (5.1, 12.4)	0.256
CT+TT	AA	696	8.6 (6.0, 14.6)	**0.021** (**0.105)**	327	11.2 (6.6, 18.5)	0.231	369	7.7 (5.6, 10.6)	**0.014** (**0.070)**
CT+TT	AG+GG	557	9.2 (5.7, 16.9)	0.307	274	12.6 (6.8, 20.4)	0.840	283	7.4 (5.1, 12.3)	0.065
rs6912200‐rs1886753										
CC	AG+GG	301	9.4 (6.0, 15.5)		131	12.0 (8.0, 19.9)		170	8.0 (5.4, 12.4)	
CC	AA	115	11.1 (6.6, 19.2)	0.330	67	13.1 (7.0, 21.3)	0.947	48	8.8 (5.5, 16.7)	0.476
CT+TT	AG+GG	903	9.1 (5.9, 15.3)	0.119	431	11.8 (6.7, 18.5)	0.226	472	7.8 (5.6, 11.8)	0.220
CT+TT	AA	344	8.6 (5.6, 16.0)	0.232	166	12.4 (6.6, 22.9)	0.703	178	7.2 (5.1, 9.5)	0.064
GC vs. CON										
rs6941539‐rs7748341										
CC	AA	527	8.5 (6.3, 14.2)		126	13.5 (8.0, 21.5)		401	8.1 (5.8, 11.9)	
CC	AG+GG	187	9.2 (5.7, 16.9)	0.508	52	16.9 (7.1, 25.2)	0.714	135	7.7 (5.7, 12.7)	0.868
CT+TT	AA	153	8.8 (5.7, 14.4)	0.714	43	14.5 (6.5, 29.4)	0.481	110	8.2 (5.7, 12.0)	0.453
CT+TT	AG+GG	107	8.1 (5.4, 14.2)	0.405	20	8.8 (6.0, 13.5)	0.134	87	8.0 (5.2, 14.3)	0.594
rs6941539‐rs72855279										
CC	GG	13	9.0 (4.5, 19.3)		4	13.5 (3.2, 52.7)		9	9.0 (5.7, 13.4)	
CC	AG+AA	700	8.6 (6.2, 15.1)	0.555	174	13.8 (8.0, 22.3)	0.584	526	8.0 (5.7, 11.9)	0.954
CT+TT	GG	14	5.6 (5.1, 10.6)	0.209	1	NA	NA	13	5.8 (4.9, 12.0)	0.434
CT+TT	AG+AA	248	8.7 (5.8, 14.2)	0.508	62	12.0 (6.4, 24.1)	0.560	186	8.2 (5.7, 12.5)	0.926
rs6941539‐rs80112640										
CC	GG	13	9.0 (4.5, 19.3)		4	13.5 (3.2, 52.7)		9	9.0 (5.7, 13.4)	
CC	AG+AA	700	8.6 (6.2, 15.1)	0.557	174	13.8 (8.0, 22.3)	0.584	526	8.0 (5.7, 11.9)	0.957
CT+TT	GG	14	5.6 (5.1, 10.6)	0.209	1	NA	NA	13	5.8 (4.9, 12.0)	0.434
CT+TT	AG+AA	250	8.7 (5.8, 14.2)	0.500	62	12.0 (6.4, 24.1)	0.560	188	8.2 (5.6, 12.5)	0.919
rs6912200‐rs72855279										
CC	GG	7	5.4 (4.7, 10.4)		2	3.7 (2.7, NA)		5	5.8 (5.4, 12.6)	
CC	AG+AA	230	9.1 (6.1, 15.0)	0.164	54	11.4 (7.6, 24.4)	0.122	176	8.7 (5.8, 13.8)	0.446
CT+TT	GG	20	8.1 (4.8, 17.4)	0.287	3	22.2 (5.4, NA)	0.316	17	8.0 (4.4, 12.9)	0.674
CT+TT	AG+AA	724	8.5 (5.9, 14.9)	0.242	185	13.9 (7.4, 22.0)	0.232	539	7.8 (5.7, 11.7)	0.628
rs6912200‐rs80112640										
CC	GG	7	5.4 (4.7, 10.4)		7	3.7 (2.7, NA)		5	5.8 (5.4, 12.6)	
CC	AG+AA	230	9.1 (6.1, 15.0)	0.164	77	11.4 (7.6, 24.4)	0.122	176	8.7 (5.8, 13.8)	0.446
CT+TT	GG	20	8.1 (4.8, 17.4)	0.287	3	22.2 (5.4, NA)	0.316	17	8.0 (4.4, 12.9)	0.674
CT+TT	AG+AA	727	8.5 (5.9, 14.7)	0.242	199	13.9 (7.4, 22.0)	0.232	542	7.8 (5.7, 11.7)	0.628
rs6939861‐rs7749023										
GG	CC	23	8.4 (5.9, 14.1)		6	5.6 (2.8, 74.6)		17	9.0 (6.8, 13.2)	
GG	AC+AA	386	8.3 (5.9, 15.1)	0.308	84	14.6 (7.1, 22.7)	0.499	302	8.0 (5.7, 11.9)	0.246
GA+AA	CC	32	6.7 (4.9, 14.1)	0.145	4	10.7 (6.1, 20.0)	0.347	28	6.2 (4.8, 13.5)	0.222
GA+AA	AC+AA	496	8.7 (6.1, 15.0)	0.341	135	13.3 (7.8, 22.9)	0.493	361	8.1 (5.7, 12.2)	0.276
rs6939861‐rs7747696										
GG	GG	24	8.7 (6.0, 14.0)		7	6.4 (2.8, 62.9)		17	9.0 (6.8, 13.2)	
GG	AG+AA	386	8.3 (5.8, 15.1)	0.301	83	14.9 (7.1, 23.0)	0.520	303	7.9 (5.7, 11.9)	0.242
GA+AA	GG	42	6.7 (4.8, 13.0)	0.114	5	13.1 (6.7, 17.8)	0.338	37	6.1 (4.7, 10.1)	0.114
GA+AA	AG+AA	490	8.8 (6.2, 15.1)	0.345	135	13.3 (7.8, 22.9)	0.510	355	8.2 (5.7, 12.3)	0.295
rs6939861‐rs72855279										
GG	GG	11	7.2 (5.4, 9.4)		3	4.7 (2.7, NA)		8	7.6 (5.6, 9.3)	
GG	AG+AA	397	8.3 (5.9, 15.1)	0.865	87	14.2 (7.1, 23.0)	0.689	310	8.0 (5.7, 12.1)	0.334
GA+AA	GG	16	7.0 (4.3, 17.4)	0.848	2	13.8 (5.4, NA)	0.738	14	7.0 (4.1, 16.8)	0.218
GA+AA	AG+AA	516	8.7 (6.1, 14.3)	0.877	138	13.2 (7.9, 22.3)	0.802	378	8.1 (5.7, 12.0)	0.368
rs6939861‐rs80112640										
GG	GG	11	7.2 (5.4, 9.4)		3	4.7 (2.7, NA)		8	7.6 (5.6, 9.3)	
GG	AG+AA	399	8.3 (5.9, 15.1)	0.869	87	14.2 (7.1, 23.0)	0.689	312	8.0 (5.7, 12.1)	0.335
GA+AA	GG	16	7.0 (4.3, 17.4)	0.848	2	13.8 (5.4, NA)	0.738	14	7.0 (4.1, 16.8)	0.218
GA+AA	AG+AA	515	8.7 (6.1, 14.3)	0.874	138	13.2 (7.9, 22.3)	0.802	377	8.1 (5.7, 12.0)	0.367
rs6939861‐rs61516247										
GG	GA+GG	384	8.2 (5.9, 15.1)		84	14.0 (6.3, 22.7)		300	8.0 (5.8, 11.9)	
GG	AA	26	8.6 (5.2, 16.3)	0.612	6	14.3 (9.3, 25.7)	0.801	20	7.6 (4.9, 12.1)	0.500
GA+AA	GA+GG	494	8.7 (6.1, 14.7)	0.991	135	13.5 (7.8, 22.2)	0.610	359	8.1 (5.7, 11.9)	0.812
GA+AA	AA	38	9.3 (5.4, 13.8)	0.791	5	11.3 (8.1, 18.0)	0.471	33	8.7 (4.6, 14.2)	0.466

Notes

*P*_corr_, *P* values after Bonferroni correction; AG, atrophic gastritis; GC, gastric cancer; CON, control; NA, not available. The results are in bold if *P* < 0.05.

### 3.7 Correlations of the single/interacting SNPs with lncRNA expression levels

The association between single lncRNA SNPs and expression of the three involved lncRNAs had not been clarified before, and thus, we investigated their expression levels in four genetic models of each SNP. Only rs7749023 was found to be associated with lncRNA expression. Its AC+AA genotype had a significantly higher level of lnc‐C6orf132‐1 when compared with CC genotype (*P* < 0.001, Table S7).

We next explored the influence of the interacting SNPs on lncRNA expression in different disease groups. The levels of lnc‐C6orf132‐1 and lnc‐LRFN‐2 were correlated with several GC‐related combinations. Notably, the AC+AA genotype of rs7749023 showed a higher level of lnc‐C6orf132‐1 in total subjects only in the presence of rs6939861 GA+AA genotype (*P* < 0.001), while no difference was observed when rs6939861 had GG genotype. When the GA+GG genotype of rs61516247 was present, the expression level of lnc‐LRFN‐2 in total subjects was higher in PGC rs6939861 GA+AA genotype than GG genotype (*P* = 0.042, Table 5).

**Table 5:** The correlations between the pairwise interacting SNPs and the expression levels of lncRNAs in serum

PGC‐lncRNA SNP genotypes		Total			Case			Control		
		Median (25%, 75%)		*P* (*P*_corr_)	Median (25%, 75%)		*P* (*P*_corr_)	Median (25%, 75%)		*P* (*P*_corr_)
		ΔCt	2^−ΔCt^		ΔCt	2^−ΔCt^		ΔCt	2^−ΔCt^	
AG vs. CON										
lnc‐C6orf132‐1										
rs9471643‐rs7749023										
GC	AA	4.06 (3.26, 4.78)	0.06 (0.04, 0.10)		4.23 (2.63, 5.88)	0.05 (0.02, 0.16)		3.36 (3.26, 4.11)	0.10 (0.06, 0.10)	
GC	AC+CC	4.86 (2.45, 5.16)	0.03 (0.03, 0.18)	0.546	4.86 (2.45, 5.16)	0.03 (0.03, 0.18)	0.649	NA	NA	0.726
GG+CC	AA	4.84 (3.22, 5.37)	0.03 (0.02, 0.11)	0.952	4.90 (3.56, 5.62)	0.03 (0.02, 0.09)	0.895	2.89 (2.65, 3.98)	0.14 (0.07, 0.16)	0.203
GG+CC	AC+CC	4.79 (3.73, 5.50)	0.04 (0.02, 0.08)	0.143	5.14 (4.15, 5.65)	0.03 (0.02, 0.06)	0.174	2.72 (2.11, NA)	0.15 (0.08, NA)	0.082
rs9471643‐rs7747696										
GC	AA	4.06 (3.25, 5.04)	0.06 (0.03, 0.11)		4.23 (2.63, 5.88)	0.05 (0.02, 0.16)		3.36 (3.16, 4.16)	0.10 (0.06, 0.11)	
GC	AG+GG	4.25 (2.47, 5.09)	0.06 (0.03, 0.18)	0.496	4.86 (2.45, 5.16)	0.03 (0.03, 0.18)	0.649	3.48 (3.31, NA)	0.09 (0.08, NA)	0.991
GG+CC	AA	4.84 (3.25, 5.48)	0.03 (0.02, 0.11)	0.977	4.95 (3.42, 5.79)	0.03 (0.02, 0.09)	0.937	2.68 (2.64, NA)	0.16 (0.05, NA)	0.318
GG+CC	AG+GG	4.78 (3.71, 5.35)	0.04 (0.02, 0.08)	0.167	4.99 (4.06, 5.59)	0.03 (0.02, 0.06)	0.184	2.91 (2.26, 3.57)	0.13 (0.09, 0.21)	0.121
rs6912200‐rs7749023										
CC	AA	4.26 (3.04, 5.04)	0.05 (0.03, 0.12)		4.95 (2.48, 5.55)	0.03 (0.02, 0.18)		3.36 (2.99, 4.20)	0.10 (0.05, 0.13)	
CC	AC+CC	4.65 (4.19, 5.36)	0.04 (0.02, 0.05)	0.410	5.04 (4.19, 5.63)	0.03 (0.02, 0.05)	0.162	NA	NA	NA
CT+TT	AA	4.47 (3.26, 5.37)	0.05 (0.02, 0.10)	0.359	4.85 (3.35, 5.87)	0.03 (0.02, 0.10)	0.323	3.31 (2.67, 3.80)	0.10 (0.07, 0.16)	0.510
CT+TT	AC+CC	4.82 (3.28, 5.40)	0.04 (0.02, 0.10)	0.354	5.03 (3.56, 5.50)	0.03 (0.02, 0.09)	0.341	3.02 (2.26, 3.63)	0.13 (0.08, 0.21)	0.219
rs6912200‐rs7747696										
CC	AA	4.29 (2.88, 5.00)	0.05 (0.03, 0.14)		4.91 (2.42, 5.72)	0.03 (0.02, 0.19)		3.74 (3.00, 4.25)	0.08 (0.05, 0.13)	
CC	AG+GG	4.65 (4.15, 5.30)	0.04 (0.03, 0.06)	0.366	5.29 (4.21, 5.55)	0.03 (0.02, 0.05)	0.148	NA	NA	0.522
CT+TT	AA	4.65 (3.25, 5.46)	0.04 (0.02, 0.10)	0.298	5.09 (3.33, 5.88)	0.03 (0.02, 0.10)	0.282	3.26 (2.66, 3.81)	0.10 (0.07, 0.16)	0.350
CT+TT	AG+GG	4.78 (3.37, 5.31)	0.04 (0.03, 0.10)	0.298	4.88 (3.58, 5.36)	0.03 (0.02, 0.08)	0.313	3.31 (2.42, 3.69)	0.10 (0.08, 0.19)	0.294
lnc‐LRFN2‐1										
rs6912200‐rs1886753										
CC	AG+GG	3.55 (2.70, 4.58)	0.09 (0.04, 0.15)		3.92 (3.39, 4.34)	0.07 (0.05, 0.10)		2.49 (2.36, NA)	0.18 (0.15, NA)	
CC	AA	3.94 (3.03, 4.63)	0.07 (0.04, 0.13)	0.509	4.11 (3.21, 4.63)	0.06 (0.04, 0.12)	0.957	3.35 (2.75, NA)	0.11 (0.07, NA)	0.333
CT+TT	AG+GG	3.29 (2.58, 4.15)	0.10 (0.06, 0.17)	0.431	3.46 (2.73, 4.39)	0.09 (0.05, 0.15)	0.229	2.37 (2.23, 3.13)	0.19 (0.11, 0.21)	0.559
CT+TT	AA	3.76 (2.86, 4.20)	0.07 (0.05, 0.14)	0.149	3.91 (3.04, 4.45)	0.07 (0.05, 0.12)	0.489	2.90 (−1.36, NA)	0.13 (0.08, NA)	0.456
GC vs. CON										
lnc‐C6orf132‐1										
rs6941539‐rs7748341										
CC	AA	4.61 (3.28, 5.09)	0.04 (0.03, 0.10)		4.89 (3.82, 5.37)	0.03 (0.02, 0.07)		3.34 (2.83, 3.76)	0.10 (0.07, 0.14)	
CC	AG+GG	5.08 (3.10, 5.73)	0.03 (0.02, 0.12)	0.655	5.35 (2.95, 5.75)	0.02 (0.02, 0.13)	0.093	NA	NA	**0.010** (**0.100)**
CT+TT	AA	4.24 (3.18, 5.42)	0.05 (0.02, 0.11)	0.729	3.86 (3.18, 5.54)	0.07 (0.02, 0.11)	0.505	3.69 (3.09, NA)	0.08 (0.05, NA)	0.379
CT+TT	AG+GG	4.79 (4.21, 5.29)	0.04 (0.03, 0.05)	0.227	5.19 (4.79, 5.55)	0.03 (0.02, 0.04)	0.466	3.23 (2.72, NA)	0.11 (0.08, NA)	0.904
rs6941539‐rs72855279										
CC	GG	NA	NA		NA	NA		NA	NA	
CC	AG+AA	4.67 (3.27, 5.35)	0.04 (0.02, 0.10)	0.686	4.90 (3.58, 5.64)	0.03 (0.02, 0.08)	0.718	3.31 (2.68, 3.64)	0.10 (0.08, 0.16)	NA
CT+TT	GG	NA	NA	NA	NA	NA	NA	NA	NA	NA
CT+TT	AG+AA	4.38 (3.42, 5.36)	0.05 (0.02, 0.09)	0.722	4.98 (3.53, 5.54)	0.03 (0.02, 0.09)	0.614	3.41 (2.81, 4.15)	0.10 (0.06, 0.14)	NA
rs6941539‐rs80112640										
CC	GG	5.01 (4.51, NA)	0.03 (0.02, NA)		NA	NA		NA	NA	
CC	AG+AA	4.65 (3.27, 5.32)	0.04 (0.03, 0.10)	0.442	4.89 (3.56, 5.68)	0.03 (0.02, 0.09)	0.718	3.31 (2.68, 3.64)	0.10 (0.08, 0.16)	NA
CT+TT	GG	NA	NA	0.873	NA	NA	NA	NA	NA	NA
CT+TT	AG+AA	4.38 (3.42, 5.36)	0.05 (0.02, 0.09)	0.424	4.98 (3.53, 5.54)	0.03 (0.02, 0.09)	0.614	3.41 (2.81, 4.15)	0.10 (0.06, 0.14)	NA
rs6912200‐rs72855279										
CC	GG	NA	NA		NA	NA		NA	NA	
CC	AG+AA	4.38 (3.36, 5.17)	0.05 (0.03, 0.10)	0.711	4.95 (4.04, 5.49)	0.06 (0.05, 0.09)	0.732	3.36 (2.99, 4.20)	0.15 (0.11, 0.19)	NA
CT+TT	GG	NA	NA	NA	NA	NA	NA	NA	NA	NA
CT+TT	AG+AA	4.74 (3.26, 5.37)	0.04 (0.02, 0.10)	0.682	4.88 (3.51, 5.58)	0.07 (0.05, 0.14)	0.787	3.29 (2.67, 3.66)	0.18 (0.11, 0.80)	NA
rs6912200‐rs80112640										
CC	GG	NA	NA		NA	NA		NA	NA	
CC	AG+AA	4.38 (3.36, 5.17)	0.05 (0.03, 0.10)	0.711	4.95 (4.04, 5.49)	0.03 (0.02, 0.06)	0.732	3.36 (2.99, 4.20)	0.10 (0.05, 0.13)	NA
CT+TT	GG	5.13 (4.77, NA)	0.03 (0.02, NA)	0.460	NA	NA	NA	NA	NA	NA
CT+TT	AG+AA	4.69 (3.26, 5.36)	0.04 (0.02, 0.10)	0.673	4.86 (3.45, 5.61)	0.03 (0.02, 0.09)	0.787	3.29 (2.67, 3.66)	0.10 (0.08, 0.16)	NA
rs6939861‐rs7749023										
GG	CC	5.28 (4.82, 5.79)	0.03 (0.02, 0.04)		5.71 (5.55, NA)	0.02 (0.02, NA)		NA	NA	
GG	AC+AA	4.29 (3.34, 5.25)	0.05 (0.03, 0.10)	0.286	4.91 (2.95, 5.68)	0.03 (0.02, 0.13)	0.668	3.64 (3.31, 4.27)	0.08 (0.05, 0.10)	NA
GA+AA	CC	5.32 (4.71, 5.46)	0.03 (0.02, 0.04)	0.739	5.35 (5.29, NA)	0.02 (0.02, NA)	0.285	NA	NA	NA
GA+AA	AC+AA	4.38 (3.22, 5.14)	0.05 (0.03, 0.11)	**<0.001** (**<0.001)**	4.82 (3.51, 5.45)	0.04 (0.02, 0.09)	0.412	2.91 (2.65, 3.33)	0.13 (0.10, 0.16)	NA
rs6939861‐rs7747696										
GG	GG	5.28 (4.82, 5.79)	0.03 (0.02, 0.04)		5.71 (5.55, NA)	0.02 (0.02, NA)		NA	NA	
GG	AG+AA	4.54 (3.33, 5.27)	0.04 (0.03, 0.10)	0.287	4.91 (2.95, 5.68)	0.03 (0.02, 0.13)	0.621	3.64 (3.31, 4.27)	0.08 (0.05, 0.10)	NA
GA+AA	GG	5.29 (4.51, NA)	0.03 (0.02, NA)	0.562	5.32 (5.29, NA)	0.03 (0.02, NA)	0.285	NA	NA	NA
GA+AA	AG+AA	4.42 (3.23, 5.17)	0.05 (0.03, 0.11)	**<0.001** (**<0.001)**	4.85 (3.56, 5.50)	0.03 (0.02, 0.09)	0.412	2.91 (2.65, 3.33)	0.13 (0.10, 0.16)	NA
rs6939861‐rs72855279										
GG	GG	NA	NA		NA	NA		NA	NA	
GG	AG+AA	4.81 (3.43, 5.35)	0.04 (0.02, 0.09)	0.700	5.09 (3.48, 5.72)	0.03 (0.02, 0.10)	0.994	3.64 (3.31, 4.27)	0.08 (0.05, 0.10)	NA
GA+AA	GG	NA	NA	NA	NA	NA	NA	NA	NA	NA
GA+AA	AG+AA	4.52 (3.24, 5.31)	0.04 (0.03, 0.11)	0.569	4.86 (3.59, 5.46)	0.03 (0.02, 0.08)	0.581	2.91 (2.65, 3.33)	0.13 (0.10, 0.16)	NA
rs6939861‐rs80112640										
GG	GG	NA	NA		NA	NA		NA	NA	
GG	AG+AA	4.81 (3.43, 5.35)	0.04 (0.02, 0.09)	0.700	5.09 (3.48, 5.72)	0.03 (0.02, 0.10)	0.994	3.64 (3.31, 4.27)	0.08 (0.05, 0.10)	NA
GA+AA	GG	5.01 (4.51, NA)	0.03 (0.02, NA)	0.873	NA	NA	NA	NA	NA	NA
GA+AA	AG+AA	4.47 (3.23, 5.23)	0.05 (0.03, 0.11)	0.561	4.85 (3.58, 5.41)	0.03 (0.02, 0.08)	0.581	2.91 (2.65, 3.33)	0.13 (0.10, 0.16)	NA
lnc‐LRFN2‐2										
rs6939861‐rs61516247										
GG	GA+GG	4.70 (3.69, 5.40)	0.04 (0.02, 0.08)		5.01 (3.07, 5.43)	0.03 (0.02, 0.12)		4.11 (3.50, 4.72)	0.06 (0.04, 0.09)	
GG	AA	4.91 (4.17, 5.49)	0.03 (0.02, 0.06)	0.462	4.91 (4.83, NA)	0.03 (0.03, NA)	0.554	NA	NA	0.963
GA+AA	GA+GG	4.01 (3.29, 5.38)	0.06 (0.02, 0.10)	**0.042** (**0.420)**	4.12 (2.95, 5.39)	0.06 (0.02, 0.13)	0.394	3.67 (3.39, 3.87)	0.08 (0.07, 0.10)	0.376
GA+AA	AA	4.39 (3.72, 5.00)	0.05 (0.03, 0.08)	0.852	4.68 (3.94, 5.01)	0.04 (0.03, 0.07)	**0.034** (**0.340)**	NA	NA	NA

Notes

*P*_corr_, *P* values after Bonferroni correction; AG, atrophic gastritis; GC, gastric cancer; CON, control; NA, not available. The results are in bold if *P* < 0.05.

## 4 DISCUSSION

In the present study, we newly found SNP interactions between PGC and its neighbor lncRNAs could enhance the susceptibility to GC/AG. Among the 15 pairwise interacting PGC‐lncRNA SNPs, five pairs were associated with AG risk and ten pairs were associated with GC risk. Furthermore, several combinations showed obvious epistasis and cumulative effects on disease risk. Based on these results, three‐way interactions were discovered when environmental factors were taken into account. We also found the interacting SNPs could affect the expression of PGC protein and involved lncRNAs. To our knowledge, this is the first time to report SNP interactions uniting protein‐coding genes and neighbor noncoding genes for the risk of gastric diseases.

As is known to all, human body is a complex organism with tens of thousands of genes, comprising encoding and noncoding genes. Each of them exerts different function and cooperates with each other to ensure coordination of normal activities of life. By exploring the interactions of genetic variation between encoding and their neighbor noncoding genes, we can know more about crosstalk of genes and obtain better understanding for the mechanism of mutual regulation. The effect of an individual SNP on disease risk was usually reported to be weak (OR < 1.5), but combination of interacting SNPs had a moderate (OR ≥ 1.5) or strong effect (OR ≥ 2) on the susceptibility to cancer.^19^, ^20^ In our previous individual study, 7 PGC SNPs and 7 lncRNA SNPs involved had been investigated and the results announced that PGC rs6939861 was associated with a weakly increased GC risk (OR = 1.32), while rs6941539 and rs6912200 had no association with any disease risk.^11^, ^12^ All the lncRNA SNPs had no effect on GC risk.^13^ In the present assembled study, we found that PGC rs6941539 combined with lnc‐C6orf‐132‐1 rs72855279 and rs80112640 had interaction ORs of 4.65 and 4.60 for GC risk; PGC rs6912200 combined with rs72855279 and rs80112640 had interaction ORs of 6.34 and 6.27; and pairwise PGC rs6939861 with lnc‐C6orf‐132‐1 rs7749023, rs7747696, rs72855279, and rs80112640 had interaction ORs of 3.87, 4.88, 5.70, and 5.76 for GC risk, respectively. All of the risk effects are strong and greater than the individual effects of related SNPs, suggesting PGC‐lncRNA SNPs could synergistically enhance the susceptibility to GC and be used as more effective markers for risk prediction. Notably, two interactions rs6939861‐rs7749023 and rs6939861‐rs7747696 also survived the Bonferroni correction, which was a strict method for multiple comparison. Given the strong significance on GC, we further calculated the population attributable fraction (PAF) to assess their clinical or public health values. The RRs were 1.19 and 1.22, and PAFs were 0.093 and 0.105, respectively. It could be drawn from the results that about 9.3% and 10.5% patients with GC in our study might be attributed to their carrying combined risk genotypes of PGC rs6939861 with lnc‐C6orf‐132‐1 rs7749023 and rs7747696. The statistics may guide larger population and indicate the potential value of combined detection of polymorphisms in PGC and its neighbor lncRNAs for GC early screening.

Among the studied polymorphisms, some SNPs made no significant contribution to GC/AG risk in the main effect analysis. For example, PGC rs6912200 CT+TT genotype showed no association with AG/GC risk compared with CC genotype (*P* = 0.637, OR = 1.06; *P* = 0.547, OR = 0.93, respectively).^11^ However, when combined with some lncRNA SNPs, obvious epistasis was observed (*P* = 0.026, OR = 1.46; *P* = 0.026, OR = 0.16, respectively). Moreover, lnc‐C6orf‐132‐1 rs7747696 was also not associated with AG/GC (*P* = 0.086, OR = 1.19; *P* = 0.670, OR = 1.05, respectively).^13^ But in the presence of PGC rs6912200 CC genotype, rs7747696 AG+AA genotype was linked to a 1.79‐fold moderate increased AG risk, which was the highest in AG‐related SNPs. On GC risk, it showed a unique strong effect (OR = 2.06) when PGC rs6939861 GA+AA genotype was present. They also demonstrated a significant cumulative effect, suggesting their cooperation with each other to confer GC susceptibility. Therefore, lnc‐C6orf‐132‐1 rs7747696 AG+GG genotype combined with PGC rs6912200 CC genotype and rs6939861 GA+AA genotype might be the superior SNP models for determination of AG/GC risk, respectively.

Apart from the host genetics, environmental factors also play critical roles in the development of gastric diseases. In this study, two environmental factors including smoking and drinking were found to have modifying effects on PGC‐lncRNA SNP interactions. The carcinogenic effect of tobacco smoke on various organs is well recognized, and it accounts for about 50% increase in GC risk.^21^, ^22^ Previously, lots of studies have investigated the interaction between other genes and smoking on GC, including TNF, Exo1, CYP1A1, IL‐10, ERCC8, GSTP1, and hTERT.^23^, ^24^, ^25^, ^26^, ^27^, ^28^, ^29^ They all suggest genetic effects of gene polymorphisms on gastric carcinogenesis can be exacerbated by cigarette smoking. Here, the interactions of PGC rs9471643 and lnc‐C6orf‐132‐1 rs7749023/rs7747696 on AG risk could also be affected by smoking, although the mechanism has not been understood. Similar to tobacco smoke, alcohol drinking is also a well‐acknowledged independent risk factor of GC, which has been reported to have interactions with genetic variations in several metabolic enzyme genes such as GSTM1 and ALDH2.^30^, ^31^ Alcohol is initially metabolized to an intermediate metabolite, acetaldehyde, which is further metabolized and eliminated from the body.^32^ Reactive oxygen species (ROSs) are produced during the generation of NADH from the conversion of ethanol to acetaldehyde by alcohol dehydrogenase and may induce gastric mucosal oxidative injury.^33^, ^34^ The modifying effects of drinking on SNP interactions for GC risk were also demonstrated in our study, which were PGC rs6941539 and lnc‐C6orf‐132‐1 rs7748341. However, it needs verification whether PGC and neighbor lncRNAs participate in alcohol metabolism.

The effects of gene polymorphisms on cancer susceptibility are often achieved by affecting the expression of its encoding protein. In the present study, we evaluated the influence of interacting SNPs on PGC protein expression in serum. With respect to PGC rs6912200 with lnc‐C6orf132‐1 rs7749023, the CT/TT+AA genotype can significantly reduce PGII level compared with the CC+AA genotype, suggesting PGC rs6912200 could affect serum PGC expression. Our research group has also found healthy subjects carried with rs6912200 CT, TT, and CT/TT variant genotypes have lower serum expression levels of PGC protein.^11^ However, the difference cannot be observed in the subjects carried with rs7749023 AC/CC genotype, indicating lnc‐C6orf132‐1 rs7749023 might counteract with rs6912200 and upregulate PGC expression. It has been revealed that SNP interactions between PGC with some host genes such as IL1B and PTPN11 may result from the alteration of PGC protein expression. PGC can also interact with polymorphisms in miRNAs that target it, including let‐7e, miR‐4795, and miR‐365b. They can bind to the 3′‐UTR region of PGC and inhibit its expression.^35^ Our study first reported the SNP interactions of PGC with its neighbor lncRNAs could also affect PGC expression. PGC protein is a well‐known marker for the differentiation of gastric epithelial cells. It serves as a proteinase involved in the digestion of protein in stomach, and its levels significantly decrease in AG and dysplasia implicating poorly differentiated cells and are more susceptible to GC.^10^ Furthermore, the serum PGII level has been proven to be promising biomarkers for diagnosis of GC and AG in recent years.^36^, ^37^ Therefore, PGC protein has close relationship with malignancy of gastric mucosa and could well recognize the risk of malignant gastric lesions. Based on the above findings, it is not difficult to speculate the possible mechanism of SNP interactions in PGC with its neighbor lncRNAs on enhancing the susceptibility to GC/AG may due to their influence on PGC expression.

The association of studied SNPs with lncRNA expression was also investigated. One single lncRNA SNP, rs7749023, was found to affect the expression level of lnc‐C6orf132‐1. Interestingly, when rs7749023 was combined with PGC rs6939861, the influence on lnc‐C6orf132‐1 expression could only be observed in the presence of rs6939861 GA+AA genotype but none in GG genotype, suggesting the expression of involved lncRNAs might also be affected by their SNP interactions. Through lncRNA expression profile and Gene Ontology (GO) analysis, we have known lnc‐C6orf132‐1 has the ability to upregulate and downregulate the expression of some oncogenes or tumor suppressor genes related to GC progression.^13^ As an important class of molecular regulators in human genomes, lncRNAs could influence the expression of nearby genes through transcription‐related process such as enhancing the activity of gene promoters, which was called cis‐acting.^38^ In our study, three interacting PGC SNPs are located in the promoter region, including rs6912200, rs6941539, and rs9471643. The neighbor lncRNAs may exert regulatory roles on PGC in cis, act with the SNPs in PGC promoters and thus demonstrate gene‐gene interactions. Further in‐depth study is needed to elucidate the molecular mechanism involved.

**Figure 1:**
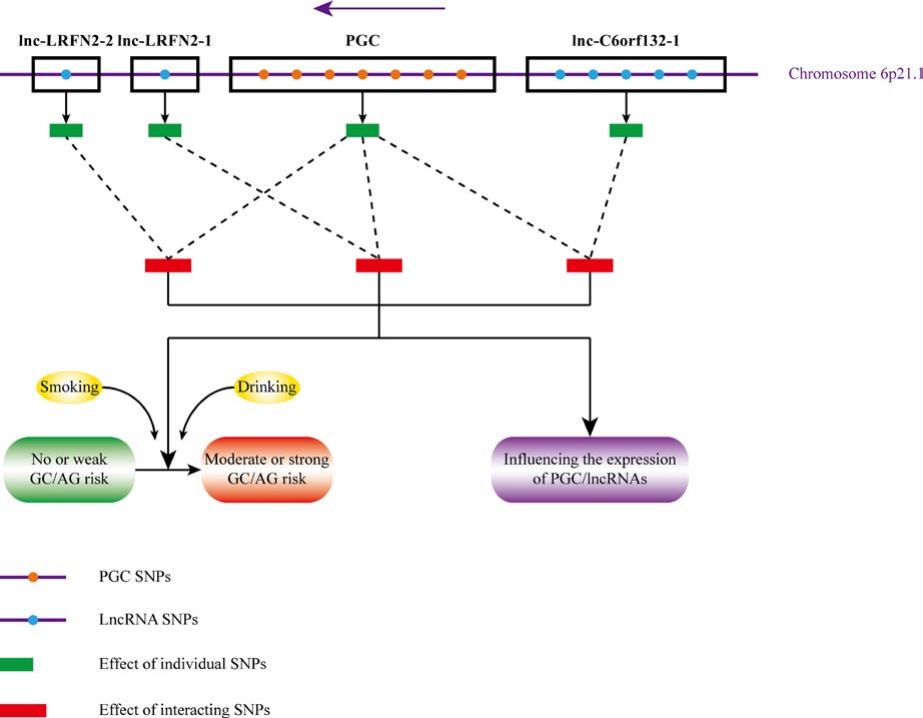
The pattern diagram of SNP interactions between PGC and its neighbor lncRNAs. A total of 14 individual SNPs are involved, including seven in PGC, five in lnc‐C6orf132‐1, one in lnc‐LRFN2‐1, and one in lnc‐LRFN2‐2. The effects of interacting SNPs modified by environmental factors could enhance the susceptibility to GC/AG and influence the expression of PGC protein and related lncRNAs

In summary (Figure 1, Table S8), we conducted a case‐control study to explore the SNP interactions between PGC and its neighbor lncRNAs for the risk of GC and AG, the modifying effects of environmental factors, and the influence of SNP interactions on the expression of PGC protein and involved lncRNAs. A total of 15 pairwise interacting PGC‐lncRNA SNPs were discovered, in which five pairs were associated with AG risk and ten pairs were associated with GC risk. By comparing the epistasis and cumulative effects, superior SNP diagnostic models for AG/GC were identified respectively. Some three‐way interactions of SNPs with smoking and drinking could also be observed. Besides, a few interacting SNPs showed correlations with the expression levels of PGC protein and related lncRNAs in serum, which might account for their gene‐gene interactions on GC/AG. Our study would provide research clues for further screening combination biomarkers uniting both protein‐coding and noncoding genes with the potential in prediction of the susceptibility to GC and its precursor.

## CONFLICT OF INTEREST

None declared.
