# Supplementary material for: SNP interactions of PGC with its neighbor lncRNAs enhance the susceptibility to gastric cancer/atrophic gastritis and influence the expression of involved molecules
Source: Cancer Med. 2018 Aug 28;7(10):5252–71. doi: 10.1002/cam4.1743 (PMC6198214; doi:10.1002/cam4.1743)
Supplement: Supplementary file 1 [file CAM4-7-5252-s001.docx]

| Table S1. The sequences of primers used in qRT-PCR | |
| --- | --- |
| Genes | Primer sequences |
| GAPDH | F: TGCACCACCAACTGCTTAG |
|  | R: GGATGCAGGGATGATGTTC |
| lnc-C6orf132-1 | F: GGGAGTGGAGAGAAGGTTTGG |
|  | R: GTTTACAGGGGAGTTGGGAGG |
| lnc-LRFN2-1 | F: GCCTATATTCTGGCCTCAGGG |
|  | R: AGGGTTCTGGAGTCGGGTTC |
| lnc-LRFN2-2 | F: GCCTGGGGTGGTGGTATAAG |
|  | R: TGTGAGAAACTGGCCCCTTC |
| Note: qRT-PCR, quantitative real-time polymerase chain reaction; F, forward primer; R, reverse primer. | |
|  |  |

| Table S2. The baseline characteristics of the subjects | | | | |
| --- | --- | --- | --- | --- |
| Variables | AG vs. CON | | GC vs. CON | |
|  | AG (%) | CON (%) | GC (%) | CON (%) |
| Total | n=810 | n=880 | n=536 | n=748 |
| Gender | *P*=0.988 | | *P*=0.655 | |
| Male | 455(56.2) | 494(56.1) | 349(65.1) | 496(66.3) |
| Female | 355(43.8) | 386(43.9) | 187(34.9) | 252(33.7) |
| Age | *P*=0.069 | | *P*=0.095 | |
| Mean±SD | 55.4±9.7 | 54.5±9.4 | 56.5±10.4 | 55.5±9.7 |
| Median | 56 | 54 | 57 | 56 |
| Range | 16-83 | 17-85 | 21-84 | 17-85 |
| *H.pylori* Infection | n=805 | n=880 | n=536 | n=748 |
|  | ***P*<0.001** | | ***P*<0.001** | |
| Positive | 474(58.9) | 247(28.1) | 276(51.5) | 209(27.9) |
| Negative | 331(41.1) | 633(71.9) | 260(48.5) | 539(72.1) |
| Smoking | n=543 | n=598 | n=218 | n=513 |
|  | *P*=0.216 | | ***P*=0.005** | |
| Ever Smoker | 171(31.5) | 209(34.9) | 112(51.4) | 206(40.2) |
| Never Smoker | 372(68.5) | 389(65.1) | 106(48.6) | 307(59.8) |
| Drinking | n=541 | n=597 | n=181 | n=512 |
|  | *P*=0.318 | | ***P*=0.003** | |
| Drinker | 124(22.9) | 152(25.5) | 75(41.4) | 151(29.5) |
| Nondrinker | 417(77.1) | 445(74.5) | 106(58.6) | 361(70.5) |
| Note: AG, atrophic gastritis; GC, gastric cancer; CON, control. The results are in bold if *P*<0.05. | | | | |

| Table S3. The interaction effects of three dimensions on the risk of gastric diseases among the pairwise interacting SNPs and smoking^a^ | | | | | | |
| --- | --- | --- | --- | --- | --- | --- |
|  |  |  |  |  |  |  |
| PGC-lncRNA SNP genotypes | | Smoking | Case | Control | *P* | OR (95%CI) |
| **AG vs. CON** | |  |  |  |  |  |
| **rs9471643-rs7749023** | |  | n=538 | n=594 |  |  |
| GC | AA | (-) | 84 | 98 |  | 1(Ref) |
| GC | AA | (+) | 30 | 39 | 0.704 | 0.90(0.51-1.57) |
| GC | AC+CC | (-) | 60 | 49 | 0.143 | 1.43(0.89-2.30) |
| GC | AC+CC | (+) | 24 | 38 | 0.309 | 0.74(0.41-1.33) |
| GG+CC | AA | (-) | 135 | 133 | 0.380 | 1.18(0.81-1.73) |
| GG+CC | AA | (+) | 63 | 78 | 0.792 | 0.94(0.61-1.47) |
| GG+CC | AC+CC | (-) | 88 | 106 | 0.877 | 0.97(0.65-1.45) |
| GG+CC | AC+CC | (+) | 54 | 53 | 0.479 | 1.19(0.74-1.92) |
|  |  |  |  |  | ***P*_interaction_=0.045(0.225**^b^**)** | |
|  |  |  |  |  | **Interaction index=3.19** | |
| **rs9471643-rs7747696** | |  | n=537 | n=595 |  |  |
| GC | AA | (-) | 75 | 93 |  | 1(Ref) |
| GC | AA | (+) | 27 | 38 | 0.668 | 0.88(0.49-1.57) |
| GC | AG+GG | (-) | 69 | 54 | 0.054 | 1.58(0.99-2.53) |
| GC | AG+GG | (+) | 26 | 39 | 0.522 | 0.83(0.46-1.48) |
| GG+CC | AA | (-) | 125 | 121 | 0.218 | 1.28(0.86-1.90) |
| GG+CC | AA | (+) | 58 | 76 | 0.813 | 0.95(0.60-1.50) |
| GG+CC | AG+GG | (-) | 99 | 119 | 0.880 | 1.03(0.69-1.55) |
| GG+CC | AG+GG | (+) | 58 | 55 | 0.272 | 1.31(0.81-2.11) |
|  |  |  |  |  | ***P*_interaction_=0.020(0.100**^b^**)** | |
|  |  |  |  |  | **Interaction index=3.81** | |
| **rs6912200-rs7749023** | |  | n=540 | n=597 |  |  |
| CC | AA | (-) | 54 | 60 |  | 1(Ref) |
| CC | AA | (+) | 21 | 29 | 0.526 | 0.81(0.41-1.57) |
| CC | AC+CC | (-) | 39 | 41 | 0.850 | 1.06(0.60-1.87) |
| CC | AC+CC | (+) | 23 | 21 | 0.581 | 1.22(0.61-2.44) |
| CT+TT | AA | (-) | 167 | 173 | 0.746 | 1.07(0.70-1.64) |
| CT+TT | AA | (+) | 71 | 89 | 0.624 | 0.89(0.55-1.44) |
| CT+TT | AC+CC | (-) | 110 | 114 | 0.762 | 1.07(0.68-1.68) |
| CT+TT | AC+CC | (+) | 55 | 70 | 0.602 | 0.87(0.52-1.45) |
|  |  |  |  |  | *P*_interaction_=0.286 | |
|  |  |  |  |  | Interaction index=0.51 | |
| **rs6912200-rs7747696** | |  | n=540 | n=598 |  |  |
| CC | AA | (-) | 50 | 56 |  | 1(Ref) |
| CC | AA | (+) | 20 | 29 | 0.460 | 0.77(0.39-1.53) |
| CC | AG+GG | (-) | 43 | 45 | 0.814 | 1.07(0.61-1.89) |
| CC | AG+GG | (+) | 23 | 21 | 0.569 | 1.23(0.61-2.48) |
| CT+TT | AA | (-) | 151 | 160 | 0.806 | 1.06(0.68-1.64) |
| CT+TT | AA | (+) | 65 | 86 | 0.513 | 0.85(0.51-1.40) |
| CT+TT | AG+GG | (-) | 127 | 128 | 0.648 | 1.11(0.71-1.75) |
| CT+TT | AG+GG | (+) | 61 | 73 | 0.799 | 0.94(0.56-1.56) |
|  |  |  |  |  | *P*_interaction_=0.436 | |
|  |  |  |  |  | Interaction index=0.61 | |
| **rs6912200-rs1886753** | |  | n=540 | n=595 |  |  |
| CC | AG+GG | (-) | 59 | 79 |  | 1(Ref) |
| CC | AG+GG | (+) | 30 | 39 | 0.921 | 1.03(0.58-1.85) |
| CC | AA | (-) | 34 | 22 | 0.024 | 2.07(1.10-3.90) |
| CC | AA | (+) | 14 | 10 | 0.161 | 1.88(0.78-4.51) |
| CT+TT | AG+GG | (-) | 206 | 207 | 0.148 | 1.33(0.90-1.97) |
| CT+TT | AG+GG | (+) | 88 | 118 | 0.995 | 1.00(0.65-1.54) |
| CT+TT | AA | (-) | 72 | 79 | 0.401 | 1.22(0.77-1.94) |
| CT+TT | AA | (+) | 37 | 41 | 0.506 | 1.21(0.69-2.11) |
|  |  |  |  |  | *P*_interaction_=0.461 | |
|  |  |  |  |  | Interaction index=1.70 | |
| **GC vs. CON** | |  |  |  |  |  |
| **rs6941539-rs7748341** | |  | n=217 | n=509 |  |  |
| CC | AA | (-) | 56 | 158 |  | 1(Ref) |
| CC | AA | (+) | 53 | 107 | 0.144 | 1.40(0.89-2.19) |
| CC | AG+GG | (-) | 20 | 48 | 0.600 | 1.18(0.64-2.15) |
| CC | AG+GG | (+) | 25 | 45 | 0.126 | 1.57(0.88-2.79) |
| CT+TT | AA | (-) | 13 | 57 | 0.201 | 0.64(0.33-1.26) |
| CT+TT | AA | (+) | 26 | 27 | 0.002 | 2.72(1.46-5.05) |
| CT+TT | AG+GG | (-) | 16 | 42 | 0.828 | 1.08(0.56-2.06) |
| CT+TT | AG+GG | (+) | 8 | 25 | 0.814 | 0.90(0.39-2.12) |
|  |  |  |  |  | *P*_interaction_=0.079 | |
|  |  |  |  |  | Interaction index=0.234 | |
| **rs6941539-rs72855279** | |  | n=217 | n=506 |  |  |
| CC | GG | (-) | 0 | 3 |  | 1(Ref) |
| CC | GG | (+) | 2 | 3 | 0.997 | NA |
| CC | AG+AA | (-) | 76 | 203 | 0.997 | NA |
| CC | AG+AA | (+) | 76 | 148 | 0.997 | NA |
| CT+TT | GG | (-) | 4 | 8 | 0.997 | NA |
| CT+TT | GG | (+) | 0 | 1 | NA | NA |
| CT+TT | AG+AA | (-) | 25 | 89 | 0.997 | NA |
| CT+TT | AG+AA | (+) | 34 | 51 | 0.997 | NA |
|  |  |  |  |  | *P*_interaction_=NA | |
|  |  |  |  |  | Interaction index=NA | |
| **rs6941539-rs80112640** | |  | n=214 | n=509 |  |  |
| CC | GG | (-) | 0 | 3 |  | 1(Ref) |
| CC | GG | (+) | 2 | 3 | 0.997 | NA |
| CC | AG+AA | (-) | 76 | 203 | 0.997 | NA |
| CC | AG+AA | (+) | 74 | 149 | 0.997 | NA |
| CT+TT | GG | (-) | 4 | 8 | 0.997 | NA |
| CT+TT | GG | (+) | 0 | 1 | NA | NA |
| CT+TT | AG+AA | (-) | 24 | 91 | 0.997 | NA |
| CT+TT | AG+AA | (+) | 34 | 51 | 0.997 | NA |
|  |  |  |  |  | *P*_interaction_=NA | |
|  |  |  |  |  | Interaction index=NA | |
| **rs6912200-rs72855279** | |  | n=213 | n=510 |  |  |
| CC | GG | (-) | 2 | 1 |  | 1(Ref) |
| CC | GG | (+) | 2 | 1 | 1.000 | 1.00(0.03-29.81) |
| CC | AG+AA | (-) | 25 | 72 | 0.160 | 0.17(0.02-2.00) |
| CC | AG+AA | (+) | 35 | 49 | 0.408 | 0.36(0.03-4.10) |
| CT+TT | GG | (-) | 2 | 10 | 0.112 | 0.10(0.01-1.71) |
| CT+TT | GG | (+) | 0 | 3 | NA | NA |
| CT+TT | AG+AA | (-) | 74 | 222 | 0.146 | 0.17(0.02-1.87) |
| CT+TT | AG+AA | (+) | 73 | 152 | 0.247 | 0.24(0.02-2.69) |
|  |  |  |  |  | *P*_interaction_=NA | |
|  |  |  |  |  | Interaction index=NA | |
| **rs6912200-rs80112640** | |  | n=213 | n=513 |  |  |
| CC | GG | (-) | 2 | 1 |  | 1(Ref) |
| CC | GG | (+) | 2 | 1 | 1.000 | 1.00(0.03-29.81) |
| CC | AG+AA | (-) | 25 | 72 | 0.160 | 0.17(0.02-2.00) |
| CC | AG+AA | (+) | 35 | 49 | 0.408 | 0.36(0.03-4.10) |
| CT+TT | GG | (-) | 2 | 10 | 0.112 | 0.10(0.01-1.71) |
| CT+TT | GG | (+) | 0 | 3 | NA | NA |
| CT+TT | AG+AA | (-) | 74 | 224 | 0.144 | 0.17(0.02-1.85) |
| CT+TT | AG+AA | (+) | 73 | 153 | 0.245 | 0.24(0.02-2.67) |
|  |  |  |  |  | *P*_interaction_=NA | |
|  |  |  |  |  | Interaction index=NA | |
| **rs6939861-rs7749023** | |  | n=209 | n=491 |  |  |
| GG | CC | (-) | 4 | 4 |  | 1(Ref) |
| GG | CC | (+) | 5 | 7 | 0.714 | 0.71(0.12-4.32) |
| GG | AC+AA | (-) | 37 | 134 | 0.078 | 0.28(0.07-1.16) |
| GG | AC+AA | (+) | 39 | 71 | 0.415 | 0.55(0.13-2.32) |
| GA+AA | CC | (-) | 4 | 14 | 0.167 | 0.29(0.05-1.69) |
| GA+AA | CC | (+) | 1 | 2 | 0.624 | 0.50(0.03-7.99) |
| GA+AA | AC+AA | (-) | 57 | 145 | 0.197 | 0.39(0.10-1.63) |
| GA+AA | AC+AA | (+) | 62 | 114 | 0.401 | 0.54(0.13-2.25) |
|  |  |  |  |  | *P*_interaction_=0.509 | |
|  |  |  |  |  | Interaction index=0.31 | |
| **rs6939861-rs7747696** | |  | n=207 | n=492 |  |  |
| GG | GG | (-) | 4 | 4 |  | 1(Ref) |
| GG | GG | (+) | 6 | 7 | 0.864 | 0.86(0.15-5.00) |
| GG | AG+AA | (-) | 36 | 135 | 0.071 | 0.27(0.06-1.12) |
| GG | AG+AA | (+) | 37 | 71 | 0.376 | 0.52(0.12-2.20) |
| GA+AA | GG | (-) | 4 | 17 | 0.108 | 0.24(0.04-1.37) |
| GA+AA | GG | (+) | 1 | 6 | 0.165 | 0.17(0.01-2.09) |
| GA+AA | AG+AA | (-) | 57 | 142 | 0.208 | 0.40(0.10-1.66) |
| GA+AA | AG+AA | (+) | 62 | 110 | 0.429 | 0.56(0.14-2.33) |
|  |  |  |  |  | *P*_interaction_=0.729 | |
|  |  |  |  |  | Interaction index=0.57 | |
| **rs6939861-rs72855279** | |  | n=209 | n=490 |  |  |
| GG | GG | (-) | 2 | 3 |  | 1(Ref) |
| GG | GG | (+) | 2 | 3 | 1.000 | 1.00(0.08-12.56) |
| GG | AG+AA | (-) | 39 | 135 | 0.369 | 0.43(0.07-2.69) |
| GG | AG+AA | (+) | 42 | 74 | 0.863 | 0.85(0.14-5.30) |
| GA+AA | GG | (-) | 2 | 8 | 0.417 | 0.38(0.04-4.00) |
| GA+AA | GG | (+) | 0 | 1 | NA | NA |
| GA+AA | AG+AA | (-) | 59 | 151 | 0.564 | 0.59(0.10-3.60) |
| GA+AA | AG+AA | (+) | 63 | 115 | 0.832 | 0.82(0.13-5.05) |
|  |  |  |  |  | *P*_interaction_=NA | |
|  |  |  |  |  | Interaction index=NA | |
| **rs6939861-rs80112640** | |  | n=208 | n=492 |  |  |
| GG | GG | (-) | 2 | 3 |  | 1(Ref) |
| GG | GG | (+) | 2 | 3 | 1.000 | 1.00(0.08-12.56) |
| GG | AG+AA | (-) | 39 | 136 | 0.365 | 0.43(0.07-2.67) |
| GG | AG+AA | (+) | 42 | 75 | 0.852 | 0.84(0.14-5.23) |
| GA+AA | GG | (-) | 2 | 8 | 0.417 | 0.38(0.04-4.00) |
| GA+AA | GG | (+) | 0 | 1 | NA | NA |
| GA+AA | AG+AA | (-) | 59 | 151 | 0.564 | 0.59(0.10-3.60) |
| GA+AA | AG+AA | (+) | 62 | 115 | 0.819 | 0.81(0.13-4.97) |
|  |  |  |  |  | *P*_interaction_=NA | |
|  |  |  |  |  | Interaction index=NA | |
| **rs6939861-rs61516247** | |  | n=209 | n=492 |  |  |
| GG | GA+GG | (-) | 37 | 130 |  | 1(Ref) |
| GG | GA+GG | (+) | 39 | 74 | 0.023 | 1.85(1.09-3.15) |
| GG | AA | (-) | 4 | 9 | 0.479 | 1.56(0.46-5.36) |
| GG | AA | (+) | 5 | 4 | 0.034 | 4.39(1.12-17.19) |
| GA+AA | GA+GG | (-) | 55 | 150 | 0.299 | 1.29(0.80-2.08) |
| GA+AA | GA+GG | (+) | 61 | 105 | 0.004 | 2.04(1.26-3.31) |
| GA+AA | AA | (-) | 6 | 9 | 0.128 | 2.34(0.78-7.01) |
| GA+AA | AA | (+) | 2 | 11 | 0.571 | 0.64(0.14-3.01) |
|  |  |  |  |  | *P*_interaction_=0.156 | |
|  |  |  |  |  | Interaction index=0.13 | |
| Note: ^a^, *P* for interaction was adjusted by gender, age and *H.pylori* infection status; ^b^, *P* values after Bonferroni correction; AG, atrophic gastritis; GC, gastric cancer; CON, control; OR, odds ratio; CI, confidence interval; NA, not available. The results are in bold if *P* for interaction <0.05. | | | | | | |
|  |  |  |  |  |  |  |

| Table S4. The interaction effects of three dimensions on the risk of gastric diseases among the pairwise interacting SNPs and drinking^a^ | | | | | | |
| --- | --- | --- | --- | --- | --- | --- |
|  |  |  |  |  |  |  |
| PGC-lncRNA SNP genotypes | | Drinking | Case | Control | *P* | OR (95%CI) |
| **AG vs. CON** | |  |  |  |  |  |
| **rs9471643-rs7749023** | |  | n=536 | n=593 |  |  |
| GC | AA | (-) | 93 | 109 |  | 1(Ref) |
| GC | AA | (+) | 20 | 30 | 0.443 | 0.78(0.42-1.47) |
| GC | AC+CC | (-) | 64 | 65 | 0.526 | 1.15(0.74-1.80) |
| GC | AC+CC | (+) | 20 | 22 | 0.852 | 1.07(0.55-2.07) |
| GG+CC | AA | (-) | 155 | 148 | 0.260 | 1.23(0.86-1.75) |
| GG+CC | AA | (+) | 42 | 62 | 0.346 | 0.79(0.49-1.28) |
| GG+CC | AC+CC | (-) | 100 | 119 | 0.938 | 0.99(0.67-1.45) |
| GG+CC | AC+CC | (+) | 42 | 38 | 0.328 | 1.30(0.77-2.18) |
|  |  |  |  |  | *P*_interaction_=0.263 | |
|  |  |  |  |  | Interaction index=2.05 | |
| **rs9471643-rs7747696** | |  | n=535 | n=594 |  |  |
| GC | AA | (-) | 81 | 104 |  | 1(Ref) |
| GC | AA | (+) | 20 | 29 | 0.709 | 0.89(0.47-1.68) |
| GC | AG+GG | (-) | 75 | 70 | 0.152 | 1.38(0.89-2.13) |
| GC | AG+GG | (+) | 20 | 23 | 0.746 | 1.12(0.57-2.17) |
| GG+CC | AA | (-) | 146 | 140 | 0.124 | 1.34(0.92-1.94) |
| GG+CC | AA | (+) | 36 | 56 | 0.460 | 0.83(0.50-1.37) |
| GG+CC | AG+GG | (-) | 110 | 128 | 0.618 | 1.10(0.75-1.62) |
| GG+CC | AG+GG | (+) | 47 | 44 | 0.219 | 1.37(0.83-2.27) |
|  |  |  |  |  | *P*_interaction_=0.076 | |
|  |  |  |  |  | Interaction index=3.11 | |
| **rs6912200-rs7749023** | |  | n=538 | n=596 |  |  |
| CC | AA | (-) | 61 | 71 |  | 1(Ref) |
| CC | AA | (+) | 14 | 17 | 0.916 | 0.96(0.44-2.10) |
| CC | AC+CC | (-) | 43 | 43 | 0.584 | 1.16(0.68-2.01) |
| CC | AC+CC | (+) | 19 | 19 | 0.680 | 1.16(0.57-2.40) |
| CT+TT | AA | (-) | 189 | 189 | 0.454 | 1.16(0.78-1.73) |
| CT+TT | AA | (+) | 47 | 75 | 0.216 | 0.73(0.44-1.20) |
| CT+TT | AC+CC | (-) | 122 | 141 | 0.974 | 1.01(0.66-1.53) |
| CT+TT | AC+CC | (+) | 43 | 41 | 0.476 | 1.22(0.71-2.11) |
|  |  |  |  |  | *P*_interaction_=0.458 | |
|  |  |  |  |  | Interaction index=1.68 | |
| **rs6912200-rs7747696** | |  | n=538 | n=597 |  |  |
| CC | AA | (-) | 56 | 68 |  | 1(Ref) |
| CC | AA | (+) | 14 | 16 | 0.882 | 1.06(0.48-2.36) |
| CC | AG+GG | (-) | 47 | 46 | 0.433 | 1.24(0.72-2.13) |
| CC | AG+GG | (+) | 19 | 20 | 0.698 | 1.15(0.56-2.37) |
| CT+TT | AA | (-) | 172 | 179 | 0.462 | 1.17(0.77-1.76) |
| CT+TT | AA | (+) | 42 | 69 | 0.256 | 0.74(0.44-1.25) |
| CT+TT | AG+GG | (-) | 140 | 152 | 0.603 | 1.12(0.73-1.71) |
| CT+TT | AG+GG | (+) | 48 | 47 | 0.431 | 1.24(0.73-2.12) |
|  |  |  |  |  | *P*_interaction_=0.339 | |
|  |  |  |  |  | Interaction index=1.95 | |
| **rs6912200-rs1886753** | |  | n=538 | n=594 |  |  |
| CC | AG+GG | (-) | 70 | 95 |  | 1(Ref) |
| CC | AG+GG | (+) | 18 | 23 | 0.864 | 1.06(0.53-2.12) |
| CC | AA | (-) | 34 | 18 | 0.004 | 2.56(1.34-4.91) |
| CC | AA | (+) | 15 | 13 | 0.274 | 1.57(0.70-3.50) |
| CT+TT | AG+GG | (-) | 234 | 236 | 0.104 | 1.35(0.94-1.92) |
| CT+TT | AG+GG | (+) | 59 | 90 | 0.611 | 0.89(0.57-1.40) |
| CT+TT | AA | (-) | 78 | 93 | 0.556 | 1.14(0.74-1.75) |
| CT+TT | AA | (+) | 30 | 26 | 0.149 | 1.57(0.85-2.88) |
|  |  |  |  |  | *P*_interaction_=0.071 | |
|  |  |  |  |  | Interaction index=3.88 | |
| **GC vs. CON** | |  |  |  |  |  |
| **rs6941539-rs7748341** | |  | n=180 | n=508 |  |  |
| CC | AA | (-) | 56 | 184 |  | 1(Ref) |
| CC | AA | (+) | 33 | 82 | 0.276 | 1.32(0.80-2.19) |
| CC | AG+GG | (-) | 19 | 64 | 0.935 | 0.98(0.54-1.77) |
| CC | AG+GG | (+) | 19 | 27 | 0.013 | 2.31(1.20-4.47) |
| CT+TT | AA | (-) | 16 | 63 | 0.570 | 0.83(0.45-1.56) |
| CT+TT | AA | (+) | 17 | 21 | 0.007 | 2.66(1.31-5.39) |
| CT+TT | AG+GG | (-) | 14 | 48 | 0.900 | 0.96(0.49-1.87) |
| CT+TT | AG+GG | (+) | 6 | 19 | 0.940 | 1.04(0.40-2.72) |
|  |  |  |  |  | ***P*_interaction_=0.049(0.490**^b^**)** | |
|  |  |  |  |  | **Interaction index=0.17** | |
| **rs6941539-rs72855279** | |  | n=180 | n=505 |  |  |
| CC | GG | (-) | 1 | 6 |  | 1(Ref) |
| CC | GG | (+) | 1 | 0 | NA | NA |
| CC | AG+AA | (-) | 74 | 242 | 0.577 | 1.84(0.22-15.49) |
| CC | AG+AA | (+) | 51 | 108 | 0.341 | 2.83(0.33-24.16) |
| CT+TT | GG | (-) | 3 | 5 | 0.326 | 3.60(0.28-46.36) |
| CT+TT | GG | (+) | 1 | 4 | 0.794 | 1.50(0.07-31.58) |
| CT+TT | AG+AA | (-) | 27 | 104 | 0.687 | 1.56(0.18-13.49) |
| CT+TT | AG+AA | (+) | 22 | 36 | 0.243 | 3.67(0.41-32.52) |
|  |  |  |  |  | *P*_interaction_=NA | |
|  |  |  |  |  | Interaction index=NA | |
| **rs6941539-rs80112640** | |  | n=177 | n=508 |  |  |
| CC | GG | (-) | 1 | 6 |  | 1(Ref) |
| CC | GG | (+) | 1 | 0 | NA | NA |
| CC | AG+AA | (-) | 74 | 242 | 0.577 | 1.84(0.22-15.49) |
| CC | AG+AA | (+) | 49 | 109 | 0.364 | 2.70(0.32-23.01) |
| CT+TT | GG | (-) | 3 | 5 | 0.326 | 3.60(0.28-46.36) |
| CT+TT | GG | (+) | 1 | 4 | 0.794 | 1.50(0.07-31.58) |
| CT+TT | AG+AA | (-) | 26 | 106 | 0.726 | 1.47(0.17-12.76) |
| CT+TT | AG+AA | (+) | 22 | 36 | 0.243 | 3.67(0.41-32.52) |
|  |  |  |  |  | *P*_interaction_=NA | |
|  |  |  |  |  | Interaction index=NA | |
| **rs6912200-rs72855279** | |  | n=176 | n=509 |  |  |
| CC | GG | (-) | 3 | 0 |  | 1(Ref) |
| CC | GG | (+) | 1 | 2 | NA | NA |
| CC | AG+AA | (-) | 29 | 86 | 0.000 | NA |
| CC | AG+AA | (+) | 22 | 34 | 0.000 | NA |
| CT+TT | GG | (-) | 1 | 11 | 0.000 | NA |
| CT+TT | GG | (+) | 1 | 2 | 0.000 | NA |
| CT+TT | AG+AA | (-) | 70 | 262 | 0.000 | NA |
| CT+TT | AG+AA | (+) | 49 | 112 | 0.000 | NA |
|  |  |  |  |  | *P*_interaction_=NA | |
|  |  |  |  |  | Interaction index=NA | |
| **rs6912200-rs80112640** | |  | n=176 | n=512 |  |  |
| CC | GG | (-) | 3 | 0 |  | 1(Ref) |
| CC | GG | (+) | 1 | 2 | NA | NA |
| CC | AG+AA | (-) | 29 | 86 | 0.000 | NA |
| CC | AG+AA | (+) | 22 | 34 | 0.000 | NA |
| CT+TT | GG | (-) | 1 | 11 | 0.000 | NA |
| CT+TT | GG | (+) | 1 | 2 | 0.000 | NA |
| CT+TT | AG+AA | (-) | 70 | 264 | 0.000 | NA |
| CT+TT | AG+AA | (+) | 49 | 113 | 0.000 | NA |
|  |  |  |  |  | *P*_interaction_=NA | |
|  |  |  |  |  | Interaction index=NA | |
| **rs6939861-rs7749023** | |  | n=172 | n=491 |  |  |
| GG | CC | (-) | 4 | 7 |  | 1(Ref) |
| GG | CC | (+) | 4 | 4 | 0.554 | 1.75(0.28-11.15) |
| GG | AC+AA | (-) | 39 | 149 | 0.231 | 0.46(0.13-1.64) |
| GG | AC+AA | (+) | 25 | 55 | 0.733 | 0.80(0.21-2.97) |
| GA+AA | CC | (-) | 3 | 12 | 0.358 | 0.44(0.08-2.55) |
| GA+AA | CC | (+) | 1 | 4 | 0.519 | 0.44(0.04-5.40) |
| GA+AA | AC+AA | (-) | 56 | 179 | 0.350 | 0.55(0.16-1.94) |
| GA+AA | AC+AA | (+) | 40 | 81 | 0.824 | 0.86(0.24-3.13) |
|  |  |  |  |  | *P*_interaction_=0.905 | |
|  |  |  |  |  | Interaction index=0.81 | |
| **rs6939861-rs7747696** | |  | n=170 | n=492 |  |  |
| GG | GG | (-) | 4 | 7 |  | 1(Ref) |
| GG | GG | (+) | 4 | 4 | 0.554 | 1.75(0.28-11.15) |
| GG | AG+AA | (-) | 39 | 150 | 0.227 | 0.46(0.13-1.63) |
| GG | AG+AA | (+) | 23 | 55 | 0.643 | 0.73(0.20-2.74) |
| GA+AA | GG | (-) | 3 | 17 | 0.185 | 0.31(0.05-1.75) |
| GA+AA | GG | (+) | 1 | 6 | 0.324 | 0.29(0.03-3.37) |
| GA+AA | AG+AA | (-) | 56 | 174 | 0.374 | 0.56(0.16-2.00) |
| GA+AA | AG+AA | (+) | 40 | 79 | 0.854 | 0.89(0.25-3.21) |
|  |  |  |  |  | *P*_interaction_=0.925 | |
|  |  |  |  |  | Interaction index=0.85 | |
| **rs6939861-rs72855279** | |  | n=172 | n=490 |  |  |
| GG | GG | (-) | 2 | 4 |  | 1(Ref) |
| GG | GG | (+) | 2 | 2 | 0.600 | 2.00(0.15-26.73) |
| GG | AG+AA | (-) | 41 | 152 | 0.485 | 0.54(0.10-3.05) |
| GG | AG+AA | (+) | 27 | 56 | 0.968 | 0.96(0.17-5.60) |
| GA+AA | GG | (-) | 2 | 7 | 0.635 | 0.57(0.06-5.78) |
| GA+AA | GG | (+) | 0 | 2 | NA | NA |
| GA+AA | AG+AA | (-) | 57 | 184 | 0.586 | 0.62(0.11-3.47) |
| GA+AA | AG+AA | (+) | 41 | 83 | 0.989 | 0.99(0.17-5.62) |
|  |  |  |  |  | *P*_interaction_=NA | |
|  |  |  |  |  | Interaction index=NA | |
| **rs6939861-rs80112640** | |  | n=171 | n=492 |  |  |
| GG | GG | (-) | 2 | 4 |  | 1(Ref) |
| GG | GG | (+) | 2 | 2 | 0.600 | 2.00(0.15-26.73) |
| GG | AG+AA | (-) | 41 | 153 | 0.480 | 0.54(0.10-3.03) |
| GG | AG+AA | (+) | 27 | 57 | 0.952 | 0.95(0.16-5.50) |
| GA+AA | GG | (-) | 2 | 7 | 0.635 | 0.57(0.06-5.78) |
| GA+AA | GG | (+) | 0 | 2 | NA | NA |
| GA+AA | AG+AA | (-) | 57 | 184 | 0.586 | 0.62(0.11-3.47) |
| GA+AA | AG+AA | (+) | 40 | 83 | 0.967 | 0.96(0.17-5.49) |
|  |  |  |  |  | *P*_interaction_=NA | |
|  |  |  |  |  | Interaction index=NA | |
| **rs6939861-rs61516247** | |  | n=172 | n=492 |  |  |
| GG | GA+GG | (-) | 39 | 146 |  | 1(Ref) |
| GG | GA+GG | (+) | 27 | 57 | 0.052 | 1.77(1.00-3.16) |
| GG | AA | (-) | 4 | 11 | 0.614 | 1.36(0.41-4.51) |
| GG | AA | (+) | 2 | 2 | 0.194 | 3.74(0.51-27.43) |
| GA+AA | GA+GG | (-) | 54 | 179 | 0.609 | 1.13(0.71-1.80) |
| GA+AA | GA+GG | (+) | 39 | 77 | 0.016 | 1.90(1.12-3.20) |
| GA+AA | AA | (-) | 5 | 12 | 0.429 | 1.56(0.52-4.69) |
| GA+AA | AA | (+) | 2 | 8 | 0.935 | 0.94(0.19-4.59) |
|  |  |  |  |  | *P*_interaction_=0.195 | |
|  |  |  |  |  | Interaction index=0.12 | |
| Note: ^a^, *P* for interaction was adjusted by gender, age and *H.pylori* infection status; ^b^, *P* values after Bonferroni correction; AG, atrophic gastritis; GC, gastric cancer; CON, control; OR, odds ratio; CI, confidence interval; NA, not available. The results are in bold if *P* for interaction <0.05. | | | | | | |
|  |  |  |  |  |  |  |

| Table S5. The interaction effects of three dimensions on the risk of gastric diseases among the pairwise interacting SNPs and *H.pylori* infection status^a^ | | | | | | |
| --- | --- | --- | --- | --- | --- | --- |
|  |  |  |  |  |  |  |
| PGC-lncRNA SNP genotypes | | *H.pylori* | Case | Control | *P* | OR (95%CI) |
| **AG vs. CON** | |  |  |  |  |  |
| **rs9471643-rs7749023** | |  | n=796 | n=869 |  |  |
| GC | AA | (-) | 59 | 147 |  | 1(Ref) |
| GC | AA | (+) | 99 | 70 | <0.001 | 3.52(2.29-5.42) |
| GC | AC+CC | (-) | 52 | 87 | 0.088 | 1.49(0.94-2.35) |
| GC | AC+CC | (+) | 82 | 37 | <0.001 | 5.52(3.38-9.03) |
| GG+CC | AA | (-) | 125 | 232 | 0.121 | 1.34(0.93-1.95) |
| GG+CC | AA | (+) | 178 | 72 | <0.001 | 6.16(4.10-9.26) |
| GG+CC | AC+CC | (-) | 90 | 161 | 0.102 | 1.39(0.94-2.07) |
| GG+CC | AC+CC | (+) | 111 | 63 | <0.001 | 4.39(2.85-6.76) |
|  |  |  |  |  | *P*_interaction_=0.300 | |
|  |  |  |  |  | Interaction index=0.63 | |
| **rs9471643-rs7747696** | |  | n=798 | n=875 |  |  |
| GC | AA | (-) | 52 | 140 |  | 1(Ref) |
| GC | AA | (+) | 92 | 66 | <0.001 | 3.75(2.40-5.88) |
| GC | AG+GG | (-) | 58 | 94 | 0.029 | 1.66(1.05-2.62) |
| GC | AG+GG | (+) | 89 | 42 | <0.001 | 5.71(3.51-9.27) |
| GG+CC | AA | (-) | 116 | 221 | 0.082 | 1.41(0.96-2.09) |
| GG+CC | AA | (+) | 165 | 69 | <0.001 | 6.44(4.21-9.84) |
| GG+CC | AG+GG | (-) | 101 | 175 | 0.031 | 1.55(1.04-2.32) |
| GG+CC | AG+GG | (+) | 125 | 68 | <0.001 | 4.95(3.21-7.64) |
|  |  |  |  |  | *P*_interaction_=0.481 | |
|  |  |  |  |  | Interaction index=0.74 | |
| **rs6912200-rs7749023** | |  | n=796 | n=870 |  |  |
| CC | AA | (-) | 33 | 83 |  | 1(Ref) |
| CC | AA | (+) | 67 | 48 | <0.001 | 3.51(2.03-6.07) |
| CC | AC+CC | (-) | 40 | 62 | 0.094 | 1.62(0.92-2.86) |
| CC | AC+CC | (+) | 58 | 23 | <0.001 | 6.34(3.38-11.90) |
| CT+TT | AA | (-) | 148 | 298 | 0.331 | 1.25(0.80-1.96) |
| CT+TT | AA | (+) | 213 | 95 | <0.001 | 5.64(3.52-9.03) |
| CT+TT | AC+CC | (-) | 104 | 185 | 0.148 | 1.41(0.88-2.26) |
| CT+TT | AC+CC | (+) | 133 | 76 | <0.001 | 4.40(2.69-7.20) |
|  |  |  |  |  | *P*_interaction_=0.311 | |
|  |  |  |  |  | Interaction index=0.61 | |
| **rs6912200-rs7747696** | |  | n=798 | n=876 |  |  |
| CC | AA | (-) | 30 | 80 |  | 1(Ref) |
| CC | AA | (+) | 64 | 47 | <0.001 | 3.63(2.07-6.38) |
| CC | AG+GG | (-) | 42 | 67 | 0.077 | 1.67(0.95-2.96) |
| CC | AG+GG | (+) | 62 | 25 | <0.001 | 6.61(3.54-12.37) |
| CT+TT | AA | (-) | 136 | 283 | 0.298 | 1.28(0.80-2.04) |
| CT+TT | AA | (+) | 193 | 89 | <0.001 | 5.78(3.55-9.43) |
| CT+TT | AG+GG | (-) | 118 | 201 | 0.066 | 1.57(0.97-2.52) |
| CT+TT | AG+GG | (+) | 153 | 84 | <0.001 | 4.86(2.96-7.98) |
|  |  |  |  |  | *P*_interaction_=0.315 | |
|  |  |  |  |  | Interaction index=0.61 | |
| **rs6912200-rs1886753** | |  | n=795 | n=873 |  |  |
| CC | AG+GG | (-) | 48 | 114 |  | 1(Ref) |
| CC | AG+GG | (+) | 84 | 56 | <0.001 | 3.56(2.21-5.74) |
| CC | AA | (-) | 25 | 32 | 0.052 | 1.86(1.00-3.46) |
| CC | AA | (+) | 42 | 16 | <0.001 | 6.23(3.20-12.15) |
| CT+TT | AG+GG | (-) | 192 | 348 | 0.164 | 1.31(0.90-1.92) |
| CT+TT | AG+GG | (+) | 240 | 128 | <0.001 | 4.45(2.99-6.64) |
| CT+TT | AA | (-) | 58 | 134 | 0.906 | 1.03(0.65-1.62) |
| CT+TT | AA | (+) | 106 | 45 | <0.001 | 5.59(3.44-9.09) |
|  |  |  |  |  | *P*_interaction_=0.250 | |
|  |  |  |  |  | Interaction index=1.87 | |
| **GC vs. CON** | |  |  |  |  |  |
| **rs6941539-rs7748341** | |  | n=532 | n=737 |  |  |
| CC | AA | (-) | 131 | 295 |  | 1(Ref) |
| CC | AA | (+) | 131 | 108 | <0.001 | 4.13(3.10-5.52) |
| CC | AG+GG | (-) | 56 | 98 | 0.240 | 1.24(0.87-1.76) |
| CC | AG+GG | (+) | 68 | 38 | <0.001 | 4.38(2.94-6.54) |
| CT+TT | AA | (-) | 49 | 78 | 0.079 | 1.40(0.96-2.04) |
| CT+TT | AA | (+) | 47 | 33 | <0.001 | 4.93(3.24-7.50) |
| CT+TT | AG+GG | (-) | 24 | 61 | 0.195 | 1.32(0.87-2.02) |
| CT+TT | AG+GG | (+) | 26 | 26 | <0.001 | 3.91(2.41-6.37) |
|  |  |  |  |  | *P*_interaction_=0.964 | |
|  |  |  |  |  | Interaction index=1.02 | |
| **rs6941539-rs72855279** | |  | n=532 | n=738 |  |  |
| CC | GG | (-) | 7 | 7 |  | 1(Ref) |
| CC | GG | (+) | 3 | 2 | 0.702 | 1.50(0.19-11.93) |
| CC | AG+AA | (-) | 180 | 386 | 0.159 | 0.47(0.16-1.35) |
| CC | AG+AA | (+) | 196 | 143 | 0.563 | 1.37(0.47-3.99) |
| CT+TT | GG | (-) | 1 | 8 | 0.080 | 0.13(0.01-1.28) |
| CT+TT | GG | (+) | 4 | 5 | 0.795 | 0.80(0.15-4.30) |
| CT+TT | AG+AA | (-) | 72 | 133 | 0.268 | 0.54(0.18-1.60) |
| CT+TT | AG+AA | (+) | 69 | 54 | 0.664 | 1.28(0.42-3.86) |
|  |  |  |  |  | *P*_interaction_=0.338 | |
|  |  |  |  |  | Interaction index=0.20 | |
| **rs6941539-rs80112640** | |  | n=529 | n=740 |  |  |
| CC | GG | (-) | 7 | 7 |  | 1(Ref) |
| CC | GG | (+) | 3 | 2 | 0.702 | 1.50(0.19-11.93) |
| CC | AG+AA | (-) | 179 | 386 | 0.156 | 0.46(0.16-1.34) |
| CC | AG+AA | (+) | 195 | 143 | 0.570 | 1.36(0.47-3.97) |
| CT+TT | GG | (-) | 1 | 8 | 0.080 | 0.13(0.01-1.28) |
| CT+TT | GG | (+) | 4 | 5 | 0.795 | 0.80(0.15-4.30) |
| CT+TT | AG+AA | (-) | 72 | 134 | 0.262 | 0.54(0.18-1.59) |
| CT+TT | AG+AA | (+) | 68 | 55 | 0.707 | 1.24(0.41-3.74) |
|  |  |  |  |  | *P*_interaction_=0.332 | |
|  |  |  |  |  | Interaction index=0.20 | |
| **rs6912200-rs72855279** | |  | n=530 | n=741 |  |  |
| CC | GG | (-) | 6 | 4 |  | 1(Ref) |
| CC | GG | (+) | 3 | 1 | 0.600 | 2.00(0.15-26.73) |
| CC | AG+AA | (-) | 67 | 118 | 0.143 | 0.38(0.10-1.39) |
| CC | AG+AA | (+) | 64 | 58 | 0.647 | 0.74(0.20-2.74) |
| CT+TT | GG | (-) | 2 | 11 | 0.036 | 0.12(0.02-0.87) |
| CT+TT | GG | (+) | 4 | 6 | 0.374 | 0.44(0.07-2.66) |
| CT+TT | AG+AA | (-) | 184 | 403 | 0.068 | 0.30(0.09-1.09) |
| CT+TT | AG+AA | (+) | 200 | 140 | 0.941 | 0.95(0.26-3.44) |
|  |  |  |  |  | *P*_interaction_=0.940 | |
|  |  |  |  |  | Interaction index=0.88 | |
| **rs6912200-rs80112640** | |  | n=530 | n=744 |  |  |
| CC | GG | (-) | 6 | 4 |  | 1(Ref) |
| CC | GG | (+) | 3 | 1 | 0.600 | 2.00(0.15-26.73) |
| CC | AG+AA | (-) | 67 | 118 | 0.143 | 0.38(0.10-1.39) |
| CC | AG+AA | (+) | 64 | 58 | 0.647 | 0.74(0.20-2.74) |
| CT+TT | GG | (-) | 2 | 11 | 0.036 | 0.12(0.02-0.87) |
| CT+TT | GG | (+) | 4 | 6 | 0.374 | 0.44(0.07-2.66) |
| CT+TT | AG+AA | (-) | 184 | 404 | 0.067 | 0.30(0.09-1.09) |
| CT+TT | AG+AA | (+) | 200 | 142 | 0.923 | 0.94(0.26-3.39) |
|  |  |  |  |  | *P*_interaction_=0.934 | |
|  |  |  |  |  | Interaction index=0.87 | |
| **rs6939861-rs7749023** | |  | n=513 | n=711 |  |  |
| GG | CC | (-) | 12 | 14 |  | 1(Ref) |
| GG | CC | (+) | 7 | 3 | 0.207 | 2.72(0.57-12.91) |
| GG | AC+AA | (-) | 85 | 213 | 0.065 | 0.47(0.21-1.05) |
| GG | AC+AA | (+) | 96 | 90 | 0.602 | 1.24(0.55-2.83) |
| GA+AA | CC | (-) | 6 | 19 | 0.103 | 0.37(0.11-1.22) |
| GA+AA | CC | (+) | 8 | 9 | 0.954 | 1.04(0.31-3.53) |
| GA+AA | AC+AA | (-) | 153 | 268 | 0.317 | 0.67(0.30-1.48) |
| GA+AA | AC+AA | (+) | 146 | 95 | 0.159 | 1.79(0.80-4.04) |
|  |  |  |  |  | *P*_interaction_=0.968 | |
|  |  |  |  |  | Interaction index=0.96 | |
| **rs6939861-rs7747696** | |  | n=512 | n=715 |  |  |
| GG | GG | (-) | 12 | 14 |  | 1(Ref) |
| GG | GG | (+) | 9 | 3 | 0.106 | 3.50(0.77-15.96) |
| GG | AG+AA | (-) | 85 | 214 | 0.063 | 0.46(0.21-1.04) |
| GG | AG+AA | (+) | 92 | 90 | 0.675 | 1.19(0.52-2.72) |
| GA+AA | GG | (-) | 6 | 26 | 0.029 | 0.27(0.08-0.87) |
| GA+AA | GG | (+) | 10 | 11 | 0.920 | 1.06(0.34-3.36) |
| GA+AA | AG+AA | (-) | 153 | 262 | 0.345 | 0.68(0.31-1.51) |
| GA+AA | AG+AA | (+) | 145 | 95 | 0.164 | 1.78(0.79-4.02) |
|  |  |  |  |  | *P*_interaction_=0.887 | |
|  |  |  |  |  | Interaction index=0.86 | |
| **rs6939861-rs72855279** | |  | n=514 | n=713 |  |  |
| GG | GG | (-) | 7 | 7 |  | 1(Ref) |
| GG | GG | (+) | 2 | 1 | 0.604 | 2.00(0.15-27.45) |
| GG | AG+AA | (-) | 90 | 221 | 0.102 | 0.41(0.14-1.19) |
| GG | AG+AA | (+) | 101 | 90 | 0.835 | 1.12(0.38-3.32) |
| GA+AA | GG | (-) | 1 | 8 | 0.080 | 0.13(0.01-1.28) |
| GA+AA | GG | (+) | 5 | 6 | 0.821 | 0.83(0.17-4.06) |
| GA+AA | AG+AA | (-) | 158 | 280 | 0.293 | 0.56(0.19-1.64) |
| GA+AA | AG+AA | (+) | 150 | 100 | 0.461 | 1.50(0.51-4.41) |
|  |  |  |  |  | *P*_interaction_=0.490 | |
|  |  |  |  |  | Interaction index=0.28 | |
| **rs6939861-rs80112640** | |  | n=513 | n=714 |  |  |
| GG | GG | (-) | 7 | 7 |  | 1(Ref) |
| GG | GG | (+) | 2 | 1 | 0.604 | 2.00(0.15-27.45) |
| GG | AG+AA | (-) | 90 | 221 | 0.102 | 0.41(0.14-1.19) |
| GG | AG+AA | (+) | 101 | 92 | 0.866 | 1.10(0.37-3.25) |
| GA+AA | GG | (-) | 1 | 8 | 0.080 | 0.13(0.01-1.28) |
| GA+AA | GG | (+) | 5 | 6 | 0.821 | 0.83(0.17-4.06) |
| GA+AA | AG+AA | (-) | 157 | 280 | 0.287 | 0.56(0.19-1.63) |
| GA+AA | AG+AA | (+) | 150 | 99 | 0.450 | 1.52(0.52-4.45) |
|  |  |  |  |  | *P*_interaction_=0.502 | |
|  |  |  |  |  | Interaction index=0.29 | |
| **rs6939861-rs61516247** | |  | n=514 | n=715 |  |  |
| GG | GA+GG | (-) | 86 | 212 |  | 1(Ref) |
| GG | GA+GG | (+) | 93 | 89 | <0.001 | 2.58(1.76-3.78) |
| GG | AA | (-) | 11 | 16 | 0.200 | 1.70(0.76-3.80) |
| GG | AA | (+) | 10 | 4 | 0.003 | 6.16(1.88-20.18) |
| GA+AA | GA+GG | (-) | 147 | 264 | 0.054 | 1.37(1.00-1.89) |
| GA+AA | GA+GG | (+) | 148 | 97 | <0.001 | 3.76(2.63-5.38) |
| GA+AA | AA | (-) | 12 | 24 | 0.578 | 1.23(0.59-2.58) |
| GA+AA | AA | (+) | 7 | 9 | 0.211 | 1.92(0.69-5.31) |
|  |  |  |  |  | *P*_interaction_=0.344 | |
|  |  |  |  |  | Interaction index=0.40 | |
| Note: ^a^, *P* for interaction was adjusted by gender and age; AG, atrophic gastritis; GC, gastric cancer; CON, control; OR, odds ratio; CI, confidence interval. | | | | | | |
|  |  |  |  |  |  |  |

| Table S6. The cumulative effects of three interacting factors on the risk of gastric diseases^a^ | | | | |
| --- | --- | --- | --- | --- |
| Number of interacting factors | Case | Control | *P* | OR (95%CI) |
| **AG vs. CON** |  |  |  |  |
| PGC rs9471643/lnc-C6orf132-1 rs7749023/smoking | | | | |
| 0 | 84 | 98 |  | 1(Ref) |
| 1 | 225 | 221 | 0.158 | 1.32(0.90-1.94) |
| 2 | 175 | 222 | 0.850 | 0.96(0.65-1.43) |
| 3 | 54 | 53 | 0.830 | 0.93(0.49-1.77) |
|  |  |  | *P*_trend_=0.736 | |
| PGC rs9471643/lnc-C6orf132-1 rs7747696/smoking | | | | |
| 0 | 75 | 93 |  | 1(Ref) |
| 1 | 221 | 213 | 0.073 | 1.44(0.97-2.15) |
| 2 | 183 | 234 | 0.821 | 1.05(0.70-1.57) |
| 3 | 58 | 55 | 0.612 | 1.19(0.61-2.31) |
|  |  |  | *P*_trend_=0.927 | |
| **GC vs. CON** |  |  |  |  |
| PGC rs6941539/lnc-C6orf132-1 rs7748341/drinking | | | | |
| 0 | 56 | 184 |  | 1(Ref) |
| 1 | 68 | 209 | 0.663 | 1.10(0.71-1.73) |
| 2 | 50 | 96 | 0.038 | 1.74(1.03-2.92) |
| 3 | 6 | 19 | 0.702 | 1.25(0.41-3.82) |
|  |  |  | *P*_trend_=0.070 | |
| Note: ^a^, *P* was adjusted by gender, age and *H.pylori* infection status; AG, atrophic gastritis; GC, gastric cancer; CON, control; OR, odds ratio; CI, confidence interval. | | | | |
|  |  |  |  |  |

| Table S7. The correlations between single lncRNA SNPs and the expression levels of lncRNAs in serum | | | | | | | | | |
| --- | --- | --- | --- | --- | --- | --- | --- | --- | --- |
| LncRNA SNP genotypes | CON | | | AG | | | GC | | |
|  | Median (25%,75%) | | *P* | Median (25%,75%) | | *P*(*P*_corr_) | Median (25%,75%) | | *P* |
|  | ΔCt | 2^-ΔCt^ |  | ΔCt | 2^-ΔCt^ |  | ΔCt | 2^-ΔCt^ |  |
| **lnc-C6orf132-1** |  |  |  |  |  |  |  |  |  |
| **rs7749023** |  |  |  |  |  |  |  |  |  |
| AA | 3.36(2.88,4.11) | 0.10(0.06,0.14) |  | 4.89(3.32,5.71) | 0.03(0.02,0.10) |  | 4.61(3.74,4.93) | 0.04(0.03,0.08) |  |
| AC | 3.02(2.26,3.63) | 0.13(0.08,0.21) | 0.186 | 4.79(3.58,5.32) | 0.04(0.03,0.08) | 0.621 | 4.77(2.56,5.78) | 0.04(0.02,0.21) | 0.745 |
| CC | NA | NA | NA | 5.50(5.32,5.71) | 0.02(0.02,0.03) | 0.232 | 4.77(4.51,NA) | 0.04(0.03,NA) | 0.472 |
| AC+CC vs. AA |  |  | 0.186 |  |  | 0.354 |  |  | 0.918 |
| CC vs. AC+AA |  |  | NA |  |  | **<0.001(<0.001)** |  |  | 0.453 |
| **rs7748341** |  |  |  |  |  |  |  |  |  |
| AA | 3.34(2.93,4.00) | 0.10(0.06,0.13) |  | 4.88(3.42,5.41) | 0.03(0.02,0.09) |  | 4.59(2.93,4.89) | 0.04(0.03,0.14) |  |
| AG | 2.72(2.11,NA) | 0.15(0.08,NA) | 0.104 | 4.99(3.70,5.65) | 0.03(0.02,0.08) | 0.668 | 5.15(4.38,NA) | 0.03(0.02,NA) | 0.308 |
| GG | NA | NA | NA | 5.68(5.50,NA) | 0.02(0.02,NA) | 0.420 | 4.77(4.51,NA) | 0.04(0.03,NA) | 0.363 |
| AG+GG vs. AA |  |  | 0.104 |  |  | 0.516 |  |  | 0.091 |
| GG vs. AG+AA |  |  | NA |  |  | 0.408 |  |  | 0.453 |
| **rs7747696** |  |  |  |  |  |  |  |  |  |
| AA | 3.36(2.78,4.19) | 0.10(0.05,0.15) |  | 4.91(3.28,5.87) | 0.03(0.02,0.10) |  | 4.63(3.58,4.98) | 0.04(0.03,0.09) |  |
| AG | 3.20(2.57,3.66) | 0.11(0.08,0.17) | 0.361 | 4.79(3.64,5.30) | 0.04(0.03,0.08) | 0.502 | 4.77(2.56,5.78) | 0.04(0.02,0.21) | 0.789 |
| GG | NA | NA | NA | 5.45(5.31,5.79) | 0.02(0.02,0.03) | 0.289 | 4.77(4.51,NA) | 0.04(0.03,NA) | 0.474 |
| AG+GG vs. AA |  |  | 0.361 |  |  | 0.323 |  |  | 0.884 |
| GG vs. AG+AA |  |  | NA |  |  | 0.261 |  |  | 0.451 |
| **rs72855279** |  |  |  |  |  |  |  |  |  |
| AA | 3.34(2.93,4.00) | 0.10(0.06,0.13) |  | 4.88(3.33,5.44) | 0.03(0.02,0.10) |  | 4.58(3.25,4.84) | 0.04(0.03,0.11) |  |
| AG | 2.72(2.11,NA) | 0.15(0.08,NA) | 0.104 | 5.24(4.15,5.73) | 0.03(0.02,0.06) | 0.312 | 5.15(5.00,NA) | 0.03(0.02,NA) | 0.271 |
| GG | NA | NA | NA | NA | NA | NA | 4.64(4.51,NA) | 0.04(0.04,NA) | 0.498 |
| AG+GG vs. AA |  |  | 0.104 |  |  | 0.312 |  |  | 0.070 |
| GG vs. AG+AA |  |  | NA |  |  | NA |  |  | 0.595 |
| **rs80112640** |  |  |  |  |  |  |  |  |  |
| AA | 3.34(2.93,4.00) | 0.10(0.06,0.13) |  | 4.86(3.32,5.40) | 0.03(0.02,0.10) |  | 4.58(3.25,4.84) | 0.04(0.03,0.11) |  |
| AG | 2.72(2.11,NA) | 0.15(0.08,NA) | 0.104 | 5.24(4.15,5.73) | 0.03(0.02,0.06) | 0.293 | 5.15(5.00,NA) | 0.03(0.02,NA) | 0.271 |
| GG | NA | NA | NA | NA | NA | NA | 4.64(4.51,NA) | 0.04(0.04,NA) | 0.498 |
| AG+GG vs. AA |  |  | 0.104 |  |  | 0.250 |  |  | 0.070 |
| GG vs. AG+AA |  |  | NA |  |  | NA |  |  | 0.595 |
| **lnc-LRFN2-1** |  |  |  |  |  |  |  |  |  |
| **rs1886753** |  |  |  |  |  |  |  |  |  |
| AA | 2.90(0.69,3.77) | 0.13(0.07,1.36) |  | 3.97(3.08,4.39) | 0.06(0.05,0.12) |  | 3.51(0.30,6.98) | 0.09(0.02,1.25) |  |
| AG | 2.36(0.14,2.60) | 0.19(0.17,2.04) | 0.727 | 3.95(3.33,4.61) | 0.06(0.04,0.10) | 0.374 | 3.76(3.22,5.39) | 0.07(0.02,0.11) | 0.152 |
| GG | 2.51(2.36,3.76) | 0.18(0.08,0.19) | 0.409 | 2.96(2.37,3.90) | 0.13(0.07,0.19) | 0.548 | NA | NA | NA |
| AG+GG vs. AA |  |  | 0.916 |  |  | 0.737 |  |  | 0.444 |
| GG vs. AG+AA |  |  | 0.179 |  |  | 0.188 |  |  | NA |
| **lnc-LRFN2-2** |  |  |  |  |  |  |  |  |  |
| **rs61516247** |  |  |  |  |  |  |  |  |  |
| GG | 3.90(3.74,4.66) | 0.07(0.04,0.08) |  | 4.99(3.29,5.43) | 0.03(0.02,0.10) |  | 4.28(3.75,5.16) | 0.05(0.03,0.07) |  |
| GA | 3.58(2.44,3.63) | 0.08(0.08,0.22) | 0.197 | 4.51(2.65,5.41) | 0.44(0.02,0.16) | 0.115 | 4.65(4.04,6.02) | 0.04(0.02,0.06) | 0.840 |
| AA | NA | NA | NA | 4.83(4.02,5.00) | 0.04(0.03,0.06) | 0.482 | 4.32(2.98,NA) | 0.07(0.02,NA) | 0.760 |
| GA+AA vs. GG |  |  | 0.089 |  |  | 0.139 |  |  | 0.785 |
| AA vs. GA+GG |  |  | NA |  |  | 0.409 |  |  | 0.742 |
| Note: *P*_corr_, *P* values after Bonferroni correction; CON, control; AG, atrophic gastritis; GC, gastric cancer; NA, not available. The results are in bold if *P*<0.05. | | | | | | | | | |

| Table S8. The summary of SNP interactions between PGC and its neighbor lncRNAs on the risk of gastric diseases | | | | | | | |
| --- | --- | --- | --- | --- | --- | --- | --- |
| Interacting PGC-lncRNA SNPs | Main effect | Epistatic effect | Cumulative effect | Interacted with smoking | Interacted with drinking | Influencing PGC protein expression | Influencing lncRNAs expression |
|  |  |  |  |  |  |  |  |
| AG-related combinations |  |  |  |  |  |  |  |
| **rs9471643-rs7749023** | No | Yes | No | Yes | No | No | No |
| **rs9471643-rs7747696** | No | Yes | No | Yes | No | No | No |
| **rs6912200-rs7749023** | No | Yes | No | No | No | Yes | No |
| **rs6912200-rs7747696** | No | Yes | No | No | No | Yes | No |
| **rs6912200-rs1886753** | No | Yes | No | No | No | No | No |
| GC-related combinations |  |  |  |  |  |  |  |
| **rs6941539-rs7748341** | No | Yes | No | No | Yes | No | Yes |
| **rs6941539-rs72855279** | No | Yes | No | No | No | No | No |
| **rs6941539-rs80112640** | No | Yes | No | No | No | No | No |
| **rs6912200-rs72855279** | No | Yes | No | No | No | No | No |
| **rs6912200-rs80112640** | No | Yes | No | No | No | No | No |
| **rs6939861-rs7749023** | Yes | Yes | No | No | No | No | Yes |
| **rs6939861-rs7747696** | Yes | Yes | Yes | No | No | No | Yes |
| **rs6939861-rs72855279** | Yes | No | Yes | No | No | No | No |
| **rs6939861-rs80112640** | Yes | No | Yes | No | No | No | No |
| **rs6939861-rs61516247** | Yes | No | Yes | No | No | No | Yes |
| Note: AG, atrophic gastritis; GC, gastric cancer. | | | | | | | |
